# Genetics-driven risk predictions leveraging the Mendelian randomization framework

**DOI:** 10.1101/gr.279252.124

**Published:** 2024-09

**Authors:** Daniel Sens, Liubov Shilova, Ludwig Gräf, Maria Grebenshchikova, Bjoern M. Eskofier, Francesco Paolo Casale

**Affiliations:** 1 Institute of AI for Health, Helmholtz Zentrum München—German Research Center for Environmental Health, 85764 Neuherberg, Germany;; 2 Helmholtz Pioneer Campus, Helmholtz Zentrum München—German Research Center for Environmental Health, 85764 Neuherberg, Germany;; 3 Friedrich-Alexander-Universität Erlangen-Nürnberg, 91054 Erlangen, Germany;; 4 School of Computation, Information and Technology, Technical University of Munich, 85748 Garching, Germany;; 5 School of Management, Technical University of Munich, 80333 Munich, Germany

## Abstract

Accurate predictive models of future disease onset are crucial for effective preventive healthcare, yet longitudinal data sets linking early risk factors to subsequent health outcomes are limited. To overcome this challenge, we introduce a novel framework, Predictive Risk modeling using Mendelian Randomization (PRiMeR), which utilizes genetic effects as supervisory signals to learn disease risk predictors without relying on longitudinal data. To do so, PRiMeR leverages risk factors and genetic data from a healthy cohort, along with results from genome-wide association studies of diseases of interest. After training, the learned predictor can be used to assess risk for new patients solely based on risk factors. We validate PRiMeR through comprehensive simulations and in future type 2 diabetes predictions in UK Biobank participants without diabetes, using follow-up onset labels for validation. Moreover, we apply PRiMeR to predict future Alzheimer's disease onset from brain imaging biomarkers and future Parkinson's disease onset from accelerometer-derived traits. Overall, with PRiMeR we offer a new perspective in predictive modeling, showing it is possible to learn risk predictors leveraging genetics rather than longitudinal data.

Large biobanks such as UK Biobank (UKB) (Sudlow et al. 2015), the German National Cohort (Wichmann et al. 2016), and others (Leitsalu et al. 2015; Abul-Husn et al. 2019), have unlocked access to extensive health metrics and risk factors in healthy individuals, enabling disease risk predictions for prevention. Yet, limited follow-up data can hinder risk predictive modeling (Pingault et al. 2018), particularly for less prevalent diseases.

Mendelian randomization (MR) is pivotal for identifying causal links between risk factors and health outcomes, utilizing genetic data across different cohorts (Smith and Ebrahim 2003; Sanderson et al. 2022). For instance, MR has elucidated the causal impact of risk factors such as cholesterol levels on cardiovascular disease, invalidating the protective role of high-density lipoprotein (HDL) cholesterol (Voight et al. 2012) and confirming the adverse effects of low-density lipoprotein (LDL) (Holmes et al. 2015). As interest grows in using MR for preventive healthcare (Glass et al. 2013; Chiolero 2018; Dixon et al. 2020; Yuan and Larsson 2020; Xu et al. 2022), we explore its potential for disease risk predictions as an alternative to longitudinal studies.

We present Predictive Risk modeling using Mendelian Randomization (PRiMeR), a novel framework for learning disease risk predictors through nonlinear functions of multiple risk factors leveraging genetic effects. To achieve this, PRiMeR utilizes risk factors and genetic data from a healthy cohort (Fig. 1A), and results from genome-wide association studies (GWAS) of diseases of interest (Fig. 1B). During training, PRiMeR fine-tunes the risk predictor function to ensure that the genetic effects on both the predictor and the disease outcome are aligned across selected genetic variants (Fig. 1C), upholding MR's foundational principles. Once trained, disease risk in new patients can be assessed solely using risk factors (Fig. 1D). It is important to note that although PRiMeR employs the MR framework for disease risk predictions, it does not constitute a test for causality.

**Figure 1.**
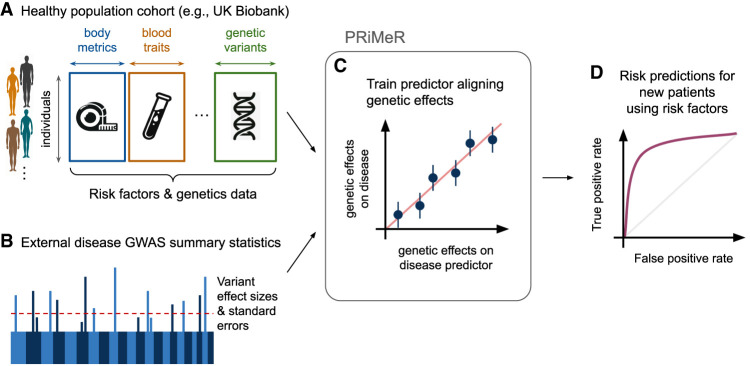
Overview of the PRiMeR framework for disease risk prediction. (*A*) PRiMeR utilizes matched health metrics and genetic data from a cohort of healthy individuals. (*B*) It integrates these with disease-specific GWAS summary statistics from an external cohort. (*C*) The framework trains risk predictors to align genetic effects with those observed in disease outcomes, maintaining adherence to two-sample MR principles. (*D*) Posttraining, the model's accuracy in predicting disease risk is evaluated, for example, through the receiver operating characteristic curve against actual follow-up disease onset data.

We validate PRiMeR's risk predictions through extensive simulations and in a type 2 diabetes (T2D) prediction task, leveraging follow-up labels for validation. Finally, we apply PRiMeR to identify a brain imaging predictor of Alzheimer's disease (AD) risk and an accelerometer-based predictor of Parkinson's disease (PD) risk.

## Results

### Predictive risk modeling using Mendelian randomization

**Figure 2.**
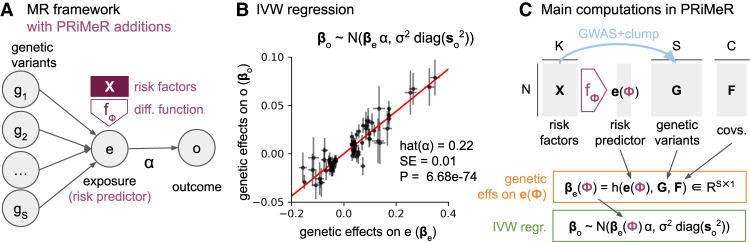
Mathematical and computational details of the PRiMeR framework. (*A*) Diagram illustrating the core MR assumptions, where genetic variants (*g*_1_, …, *g*_*S*_) influence exposure (*e*), which in turn affects outcome (*o*) with directional effect *α*. Additions unique to PRiMeR are highlighted in purple: a risk predictor is computed as a differentiable function $f_{\phi }$ (parametrized by *ϕ*) of risk factors *X*. (*B*) Illustration of IVW regression, where genetic variant effects on outcome (*β*_*o*_) are regressed on the aggregate risk predictor (*β*_*e*_), accounting for their standard errors (*s*_*o*_). (*C*) Main computations in PRiMeR, including computation of the risk predictor *e*(*ϕ*), the estimation of genetic effects on the risk predictor *β*_*e*_(*ϕ*), and the computation of the IVW regression loss. The function *h*(*e*(*ϕ*), *G*, *F*) returns marginal regression weights of each variant *G*_:*s*_ on *e*(*ϕ*) accounting for covariates *F*. As all these steps are differentiable, $f_{\phi }$ can be learned through gradient-based optimization of the IVW regression loss.

Traditionally, two-sample MR utilizes GWAS summary statistics of a risk factor (exposure, e.g., LDL cholesterol) and a disease (outcome, e.g., cardiovascular disease) from different cohorts to assess the directional effect of the exposure on the outcome (Fig. 2A). MR operates under the premise that, given essential assumptions, if an exposure causally influences an outcome, then the effects of exposure-associated variants on the outcome should be directly proportional to their effects on the exposure, with the slope of this proportionality quantifying the directional effect (Sanderson et al. 2022). Technically, for *S* independent exposure-associated variants, the directional effect $\hat{\alpha }$ is estimated through inverse variance weighting (IVW) regression (Burgess et al. 2013), where the genetic effects on the outcome (*β*_*o*_ ∈ *R*^*S*^) are regressed on the genetic effects on the exposure (*β*_*e*_ ∈ *R*^*S*^), while accounting for their standard errors (*s*_*o*_ ∈ *R*^*S*^) (Fig. 2B). A critical assumption of MR is the absence of horizontal pleiotropy; that is, exposure-associated genetic variants must affect the outcome solely via the exposure, without affecting alternative pathways (Verbanck et al. 2018).

In this work, we investigate the use of the two-sample MR framework to learn disease risk predictors, enabling predictive modeling when longitudinal data are missing or scarce. To clarify how this could be feasible, we provide an illustrative example: Suppose directional effects ${\hat{\alpha }}_{1},\ldots ,\;{\hat{\alpha }}_{K}$ from *K* candidate risk factors *x*_1_, …, *x*_*K*_ on a disease outcome are determined through two-sample MR. These effects can be aggregated to construct a linear risk predictor $f(x)=\underset{k\in C}{\sum }{\hat{\alpha }}_{k}x_{k}$, where *C* represents the set of significant directional effects.

With PRiMeR, we extend this concept to learn nonlinear risk predictors combining multiple risk factors, leveraging individual-level data from a genetic cohort of healthy individuals and disease-specific GWAS summary statistics. To do so, we introduce a differentiable function *f* parametrized by *ϕ* aggregating multiple risk factors into a single risk predictor (Fig. 2A). The predictor is then fine-tuned to optimize the IVW regression. Briefly, for *N* individuals, *K* risk factors *X* ∈ *R*^*N*×*K*^, *C* covariates *F* ∈ *R*^*N*×*C*^, and *S* independent genetic variants *G* ∈ *R*^*N*×*S*^ associated with at least one of the *K* risk factors, the IVW regression loss can be computed as follows (Fig. 2C):

1. Compute aggregate risk predictor *e*(*ϕ*) ∈ *R*^*N*×1^ from *X* using $f_{\phi }$.
2. Compute genetic effects *β*_*e*_(*ϕ*) ∈ *R*^*S*×1^ on the aggregate risk predictor as the marginal regression weights of each variant *G*_:*s*_ on *e*(*ϕ*) accounting for covariates *F*. This step mirrors the risk factor GWAS step in standard MR.
3. Compute IVW regression loss based on risk predictor genetic effects *β*_*e*_(*ϕ*), and disease outcome statistics *β*_*o*_ and *s*_*o*_; that is, $L_{\mathrm{IVW}}(\phi ,\;\alpha ,\sigma^{2})=-\log\;N({\text{β}}_{o}|{\text{β}}_{e}(\phi )\alpha ,\sigma^{2}\mathrm{diag}(s_{o}^{2}))$.

As all these steps are differentiable, *ϕ* can be learned through gradient-based optimization of the IVW loss (Methods). To select independent risk factor-associated variants for our analyses, we performed univariate GWAS analyses for each risk factor followed by a multivariate clumping procedure (Methods). We considered the following nonlinear function of risk factors:

$$f_{\phi }(x)=\overset{K}{\underset{k=1}{\sum }}a_{k}\;g(b_{k}x_{k}+c_{k}),$$

with parameters *ϕ* = {*a*_1_, …, *a*_*K*_, *b*_1_, …, *b*_*K*_, *c*_1_, …, *c*_*K*_}, where *g* is a nonlinear increasing warping function, a choice that enables modeling potential nonlinearities while being simple and clinically plausible. Such a shape function is commonly used in risk prediction as it captures the scenario where contributions from single risk factors remain minimal until a critical threshold and then escalate (Wainberg et al. 2019; Liang et al. 2020; Zhao et al. 2023).

### Validation of PRiMeR using simulated data

We evaluated the proposed PRiMeR framework through a series of simulations derived from UKB, encompassing 309,846 unrelated European individuals. We focused on 26 blood traits observed as potential risk factors in healthy individuals, simulating scenarios where subsets of these traits affect future health outcomes. Importantly, the aggregate risk was simulated as a linear combination of contributions from these factors, each transformed by a nonlinear increasing warping function to represent contributions that activate beyond specific thresholds (Methods). Our simulation framework allowed us to examine the efficacy of PRiMeR under various conditions, including the presence of horizontal pleiotropy and varying degrees of risk factor influence on outcome variance.

We compared PRiMeR with its linear variant (PRiMeR-LIN) and linear risk predictors based on two-sample univariate and multivariable Mendelian randomization (UVMR-based and MVMR-based, respectively; Methods). Beyond MR-derived models, we included a longitudinal reference model (LRM) trained directly on individual-level follow-up labels as a performance benchmark (Methods). Our evaluation maintained a strict two-sample framework, preventing any overlap between the cohorts used for determining genetic effects on risk factors and outcomes. We measured the accuracy of the risk predictions for all methods using Spearman's correlation coefficient, comparing estimated risk scores versus simulated ones in a held-out validation set. To ensure the calibration of our evaluation procedure, we verified models’ performance was equivalent to random chance in control simulations without a directional effect (Supplemental Fig. A1).

**Figure 3.**
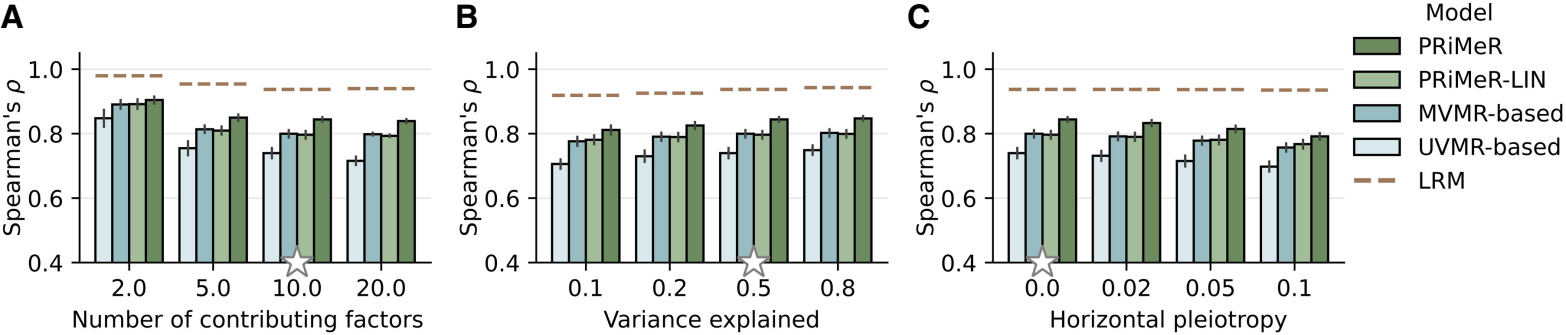
Assessment of disease risk prediction accuracy using simulated data. Comparison of model accuracy in recovering the simulated aggregate risk factor measured by Spearman's correlation coefficient. Compared are PRiMeR, its linear variant (PRiMeR-LIN), a predictor based on multivariable MR (MVMR-based), a predictor based on univariate MR (UVMR-based), and the supervised model accessing individual-level follow-up labels (LRM; Methods), varying the number of contributing risk factors (*A*), the fraction of outcome variance explained by the risk factors (*B*), and the fraction of outcome variance explained by horizontal pleiotropy (*C*). Stars denote standard values held constant while other parameters were varied. Error bars indicate standard errors across 10 replicate experiments.

The findings from our simulations underscore the robustness and versatility of PRiMeR across a wide range of scenarios. Specifically, PRiMeR's performance in estimating risk remained stable when increasing the number of causal risk factors (Fig. 3A). This superior performance persisted across different values of the variance explained by the risk factors (Fig. 3B) and when simulating horizontal pleiotropy (Fig. 3C; Methods). We also assessed the robustness of PRiMeR across differently transformed contributions of single risk factors (Supplemental Fig. A2; Methods) and varying numbers of observed genetic variants strongly associated with the risk factors (Supplemental Fig. A3; Methods).

Finally, we note that although the LRM offers the highest predictive accuracy, PRiMeR's performance can be competitive if follow-up data are sparse (Supplemental Fig. A4). Collectively, these results highlight PRiMeR's potential as a powerful tool for predictive modeling when follow-up labels are scarce.

### Validation of PRiMeR in predicting 5-year type 2 diabetes risk

**Figure 4.**
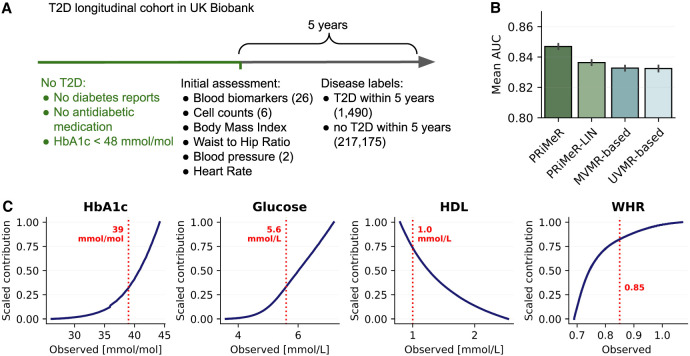
Validation of PRiMeR in predicting 5-year T2D risk. (*A*) Schematic representation of UKB T2D cohort, showing the inclusion criteria, the 37 risk factors included in our analysis, and the definition of the 5-year T2D onset labels. (*B*) Comparison of the mean area under the receiver operating characteristic curve (AUC) scores for 5-year T2D onset labels obtained using PRiMeR, its linear variant (PRiMeR-LIN), a predictor based on multivariable MR (MVMR-based) and univariate MR (UVMR-based). Error bars denote standard errors across 50 random train/test splits (Methods). (*C*) Scaled contributions to risk learned by PRiMeR as function of observed values for glycated hemoglobin (HbA1c), glucose, HDL, and waist-to-hip ratio (WHR). Risk reference thresholds are annotated in red.

Next, we evaluated the prediction accuracy of PRiMeR in a real-world setting, considering a T2D data set derived from the UKB data set. Specifically, we aimed to predict 5-year T2D risk by leveraging risk factors and genetic data from 218,665 UKB individuals with no reported T2D at the initial assessment (Methods), and external GWAS summary statistics for T2D (Mahajan et al. 2018). As input risk factors, we used 37 traits previously linked to diabetes risk (Edlitz and Segal 2022), including metabolic, anthropometric, and cardiovascular metrics (Fig. 4A). We used 6077 independent genetic variants associated with at least one of the risk traits at the genome-wide significance level (*P* < 5 × 10^−8^; Methods).

PRiMeR outperformed baseline MR methods, achieving an average AUC of 0.847 (±0.002) against 0.836 (±0.002) obtained using the MVMR-based predictor (*P* < 10^−4^) (Fig. 4B; Supplemental Fig. A5). Additionally, we evaluated MR-based model predictions against a polygenic risk score (PRS) model that relies exclusively on genetic data for predictions (Thompson et al. 2024; Methods), unlike MR-based models which use risk factors for prediction. Notably, the PRS model markedly underperformed compared to MR-based models, recording an AUC of 0.647 (±0.002) (Supplemental Fig. A5). Although a supervised reference model expectedly yielded the best performance when trained on full individual-level follow-up data, PRiMeR demonstrated competitiveness in scenarios with low numbers of follow-up labels (Supplemental Fig. A4).

The risk predictor derived from PRiMeR robustly aligns with established clinical knowledge, underscored by its correlation with individual factors (Supplemental Fig. A6; Edlitz and Segal 2022). Notably, the nonlinear relationships identified by PRiMeR align with clinical expectations; for example, the risk contributions from glycated hemoglobin and glucose show significant increases nearing clinical risk thresholds (Fig. 4C). Overall, these results showcase the accuracy and clinical plausibility of risk predictors learned through PRiMeR in a real data setting.

### Application of PRiMeR to predict 5-year Alzheimer's disease risk from brain imaging biomarkers

We applied PRiMeR to identify imaging biomarkers predictive of 5-year AD risk focusing on 31,552 unrelated European individuals in the UKB with brain imaging data. As imaging risk factors, we considered 70 subcortical and gray matter volume traits from T1 MRI having at least five independent genome-wide significant signals, for a total of 353 independent genetic variants associated with at least one of these traits. As external AD GWAS results, we used AD GWAS summary statistics from Wightman et al. (2021).

**Figure 5.**
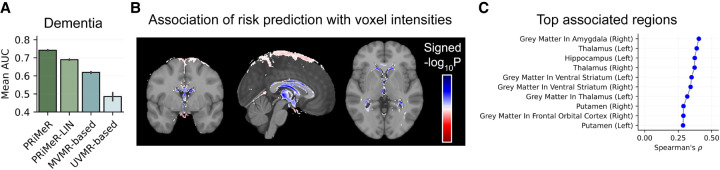
Application of PRiMeR to predict 5-year AD risk. (*A*) Comparative performance of PRiMeR against baseline MR models using average AUC for 5-year AD predictions using follow-up labels. (*B*) Heatmap of the signed $-{log}_{10}$ *P-*value of association between voxel intensities and the AD risk predictor scores, overlayed on the MNI152 template (Miller et al. 2016; Alfaro-Almagro et al. 2018; https://www.bic.mni.mcgill.ca/ServicesAtlases/ICBM152NLin6). Areas where increased risk predictor scores correlate with significant increased (decreased) voxel intensities are highlighted in red (blue) (Bonferroni-adjusted *P* < 0.05). (*C*) Spearman's correlation coefficients between the AD risk predictor and individual MRI traits in the validation set. Results for the top 10 associated regions are displayed, with associations for all analyzed regions available in Supplemental Figure A7.

All multivariable MR models exceeded the performance expected by chance (Fig. 5A), with PRiMeR achieving markedly higher accuracy compared to linear counterparts (PRiMeR AUC at 0.741 ± 0.003 vs. PRiMeR-LIN at 0.690 ± 0.003 vs. MVMR-based at 0.629 ± 0.004) (Fig. 5B). A thorough analysis of the key imaging features pinpointed by PRiMeR for AD predictions underscored their correlation with reductions in gray matter and subcortical volume across various regions (Supplemental Fig. A7), particularly in the midbrain (Fig. 5B,C), consistent with known AD pathology (Knopman et al. 2021). Overall, these results underscore PRiMeR's effectiveness in utilizing genetic data for accurate risk prediction in the context of diseases with lower prevalence, such as AD.

### Application of PRiMeR to predict 5-year Parkinson's disease risk from accelerometer features

We applied PRiMeR to learn the risk of 5-year PD, focusing on 69,670 unrelated European individuals in the UKB with accelerometer data. As risk factors, we considered 38 accelerometer-derived biomarkers having at least one independent genome-wide significant signal (Methods), and we considered external PD GWAS summary statistics from the FinnGen cohort (Kurki et al. 2023).

PRiMeR and PRiMeR-LIN achieved the highest accuracy (AUC of 0.787 ± 0.003 and 0.784 ± 0.003, respectively). In contrast, the MVMR-based and UVMR-based predictors showed significantly lower accuracies with AUCs of 0.521 ± 0.006 and 0.519 ± 0.006, respectively (Supplemental Fig. A8). When compared to a PRS model, PRiMeR demonstrated superior predictive performance (AUC of 0.787 ± 0.003 vs. 0.624 ± 0.001) (Supplemental Fig. A8). The analysis of key accelerometer features revealed their strong association with sleep duration and physical activity levels (Supplemental Fig. A9), confirming recent findings (Schalkamp et al. 2023).

## Discussion

In this study, we demonstrate that the two-sample MR framework can be extended to enable disease risk predictions using genetic information, without relying on longitudinal data. We introduce PRiMeR, a method for genetics-based risk predictions that leverages results from disease GWAS as supervisory signals for training risk predictors. The introduced approach is especially valuable given that genetic biobanks boast extensive health metrics but can lack longitudinal disease onset data for specific diseases, especially for those with lower incidence rates.

We validated PRiMeR through simulations and applications to predict diabetes from cardiovascular health indicators, AD from brain imaging biomarkers, and PD from accelerometer data. In simulations, PRiMeR outperformed baseline models and demonstrated robustness to horizontal pleiotropy, where genetic variants influence outcomes through alternative pathways, creating outliers in the regression of genetic effects central to two-sample MR. In real data applications, PRiMeR effectively recapitulated several established risk factors. In the T2D application, higher risk correlated with higher body mass index and waist-to-hip ratio (Belkina and Denis 2010; Burhans et al. 2018), lower levels of sex hormone-binding globulin (SHBG) (Huang et al. 2023), and an imbalance in cholesterol levels (Lewis and Steiner 1996; Sparks et al. 2012; Vergès 2015)—that is, higher LDL and lower HDL (Supplemental Fig. A6). In the brain imaging application, lower volumes in the amygdala, thalamus, and hippocampus correlated with higher AD risk (Supplemental Fig. A7), aligning with known disease pathogenesis (Vereecken et al. 1994; de Jong et al. 2008; Poulin et al. 2011). Finally, lower sleep duration and reduced physical activity were linked to increased PD risk (Supplemental Fig. A9), confirming known early stage symptoms (Xu et al. 2010; Lysen et al. 2019; Schalkamp et al. 2023).

Despite its advantages, PRiMeR is not without limitations. PRiMeR requires genetic cohorts for risk factors and outcomes to be sampled from the same population, and failure to meet these criteria may lead to challenges due to variations in linkage disequilibrium patterns (The International HapMap Consortium et al. 2007), necessitating the integration of robust instrument selection strategies, such as variant fine-mapping (Cai et al. 2023). Although the simple nonlinearity implemented in PRiMeR enables interpretability, it may fail to capture more complex relationships between risk factors. Although extensions to more complex parametric forms or flexible neural network functions would mitigate this, managing overfitting will represent a key challenge. Future work to address this may involve extending PRiMeR's Bayesian framework by integrating recent developments in deep probabilistic models (Kingma and Welling 2014; Nikolentzos et al. 2023). Furthermore, incorporating explicit mechanisms to counter weak instrument bias (Wang and Kang 2022) and horizontal pleiotropy (Sanderson et al. 2022) are critical areas for further development.

Finally, although our focus has been on predicting disease risk without reliance on longitudinal data, utilizing a causal inference framework for disease prediction may provide a viable method to mitigate confounding in longitudinal data sets (Pingault et al. 2018), potentially enhancing the generalization of risk predictors across different data sets. The potential of PRiMeR to facilitate this exploration opens exciting avenues for future research, particularly as more cohorts with deeper phenotype data become available.

As we look to the future, we identify three key areas where PRiMeR can make a significant impact. It offers promising solutions for diseases with low prevalence and well-developed GWAS, such as Alzheimer's, Parkinson's, amyotrophic lateral sclerosis, and bipolar disorder. These applications are critical where traditional longitudinal analyses are often limited by small sample sizes, particularly for specialized biomarker modalities—for example, only 19 individuals developed AD within 5 years in the T1 brain MRI cohort in our analysis. Additionally, PRiMeR has potential applications in underdiagnosed diseases such as attention deficit hyperactivity disorder, depression, and fatty liver disease, all of which have robust GWAS but lack reliable diagnostic labels for longitudinal analysis. Finally, we are poised to extend PRiMeR's application to molecular genetic data sets, such as bulk and single-cell expression quantitative trait loci data sets (Lonsdale et al. 2013; van der Wijst et al. 2020; Yazar et al. 2022), where longitudinal information is typically not available.

## Methods

### Predictive risk modeling utilizing Mendelian randomization

#### Two-sample Mendelian randomization and inverse variance weighting

Two-sample MR leverages summary statistics of GWAS of a risk factor (exposure) and a health outcome to infer the causal effect of the exposure on the outcome. Assuming *S* independent genetic variants associated with the analyzed exposure, this can be estimated through the IVW regression (Burgess et al. 2013):

(1) $${\text{β}}_{o}∼N({\text{β}}_{e}\alpha ,\sigma^{2}\mathrm{diag}(s_{o}^{2})),$$

where N denotes the multivariate normal distribution, *β*_*o*_ ∈ *R*^*S*^ denotes the variant effects on the outcome, *s*_*o*_ ∈ *R*^*S*^ is the standard errors, *β*_*e*_ ∈ *R*^*S*^ is the variant effects on the exposure, *α* is the regression slope, and *σ*^2^ is the variance of the regression error. Within this framework, the causal effect of the exposure on the outcome is the maximum likelihood estimator of the regression slope *α*, that is, $\hat{\alpha }=\;\frac{\sum_{i=1}^{S}w_{i}\;{\text{β}}_{e,i}\;{\text{β}}_{o,i}\;}{\sum_{i=1}^{S}w_{i}\;{\left({\text{β}}_{e,i}\right)}^{2}}$ with standard error $\mathrm{SE}(\hat{\;\alpha \;})=\frac{1}{\sqrt{\sum_{i=1}^{S}w_{i}\;{\left({\text{β}}_{e,i}\right)}^{2}}}$, where $w_{i}=\frac{1}{{\left(s_{o,i}\right)}^{2}}$. MR is an instrumental variable analysis method (Angrist et al. 1996), using the genetic variants associated with the exposure as instruments. As such, it relies on key assumptions (Sanderson et al. 2022): (1) The chosen instruments are robustly associated with the exposure; (2) the instruments are independent of any confounders that may influence both the exposure and the outcome; and (3) the instruments influence the outcome only through the exposure, that is, no horizontal pleiotropy. Moreover, for the causal effect estimate to be valid, the exposure and outcome statistics need to be estimated on independent cohorts sampled from the same population (Zhao et al. 2019).

#### Predictive risk modeling utilizing Mendelian randomization

In classical two-sample MR, *β*_*e*_ is retrieved from the GWAS of a single risk factor. However, given access to individual-level data and multiple risk factors, it is feasible to define an aggregate risk factor as a function of these factors and compute *β*_*e*_ by regressing genetic instruments against this aggregate risk factor. Importantly, if the function $f_{\phi }$ parametrized by *ϕ* is differentiable, the corresponding genetic effects on the aggregate risk factor *β*_*e*_(*ϕ*) are also differentiable, enabling the learning of an aggregate risk function $f_{\phi }$ by directly optimizing the IVW regression loss through gradient descent. For *N* individuals, *K* risk factors *X* ∈ *R*^*N*×*K*^, *C* covariates *F* ∈ *R*^*N*×*C*^, and *S* independent genetic variants *G* ∈ *R*^*N*×*S*^, each associated with at least one of the *K* risk factors, the IVW regression loss can be computed as

$$L_{\mathrm{IVW}}(\phi ,\;\alpha ,\;\sigma^{2})=-\log N\;({\text{β}}_{o}\;|\;{\text{β}}_{e}(\phi )\;\alpha ,\;\sigma^{2}\;\mathrm{diag}(s_{o})),\;$$

where the genetic effects of the aggregated risk factor *β*_*e*_(*ϕ*) are computed as the marginal regression weights of each variant *G*_:,1_, …, *G*_:,*S*_ on $f_{\phi }(X)$ accounting for covariates *F* (Supplemental Information). As $L_{\mathrm{IVW}}$ is fully differentiable in *ϕ*, *α*, σ^2^, the predictor function $f_{\phi }$ is end-to-end trainable. Regarding the analytical form of *f*, we opted for a linear combination of nonlinear increasing warping functions of single risk factors:

$$f_{\phi }(X)\;=\;\overset{K}{\underset{k=1}{\sum }}a_{k}\;\mathrm{ELU}(b_{k}\;X_{k}\;+\;c_{k}),$$

with parameters *ϕ* = {*a*_1_, …, *a*_*K*_, *b*_1_, …, *b*_*K*_, *c*_1_, …, *c*_*K*_} and where ELU(·) is the exponential linear unit function (Clevert et al. 2015). This formulation assumes contributions from single risk factors remain minimal until a critical threshold is reached, after which they escalate (Wainberg et al. 2019; Liang et al. 2020; Zhao et al. 2023). Note that PRiMeR reduces to multivariable MR when selecting a linear function for $f_{\phi }$ (Supplemental Information; Burgess and Thompson 2015; Sanderson et al. 2020), underscoring the robust foundation and adaptability of our approach. An overview of related methods to PRiMeR is detailed in Supplemental Information.

#### Bayesian model and optimization

To enhance PRiMeR's robustness in scenarios with a limited number of genetic variants robustly associated with the analyzed risk factors, we implemented a Bayesian inference approach. This involved introducing priors over the parameters *ϕ* and optimizing the log marginal likelihood of the IVW model. For parameters where analytical integration was infeasible, mean-field variational inference was utilized to derive the evidence lower bound (ELBO). Optimization of the ELBO was achieved through gradient descent using the Adam optimizer, incorporating the reparametrization trick to enable backpropagation through the expectation term of the ELBO. This approach aligns with standard practices in variational inference methods that leverage gradient descent (Kingma and Welling 2014; Ranganath et al. 2014; Engelmann et al. 2024). The learning rate for all experiments was fixed at 0.01, and we consistently applied gradient clipping with a norm bound of one while training for 1000 epochs. Risk predictions were obtained as the mean of the variational posterior of the model. Comprehensive details on our Bayesian model and variational inference procedure can be found in Supplemental Information. Finally, we note that prior to all experiments, risk factors were normalized using a rank-inverse Gaussian transformation, a widely used phenotype transformation for GWAS analyses (McCaw et al. 2020). Our PRiMeR framework was implemented in PyTorch (Paszke et al. 2019).

#### Selection of genetic variants

To identify genetic variants associated with risk factors, we first conducted a univariate GWAS for each risk factor followed by a multivariate clumping procedure. GWAS analysis utilized linear regression via GCTA (fastGWA-lr functionality) (Jiang et al. 2019), adjusting for sex, age, UKB array type, and the top 20 genetic principal components. Adjusting for the top 20 genetic principal components is a standard practice to correct for population structure in genetic analyses of unrelated Europeans (Price et al. 2006). After GWAS, we applied clumping on the minimum *P*-value statistics across all traits using PLINK (Purcell et al. 2007), with parameters fixed to a *P*-value threshold of 5 × 10^−8^, an *r*^2^ linkage disequilibrium cutoff of 0.05, and a clumping window of 5000 kb, following Zhu et al. (2018). This procedure ensured that selected variants are approximately independent and associated with at least one of the risk factors at genome-wide significant level (*P* < 5 × 10^−8^).

#### Comparison models

In our study, we assess the performance of PRiMeR in comparison to predictive models based on univariate Mendelian randomization (UVMR-based) and multivariate Mendelian randomization (MVMR-based). Both UVMR-based and MVMR-based prediction methods apply a linear risk prediction function $f(X)=\overset{K}{\underset{k=1}{\sum }}a_{k}X_{:,k}$, where *X* represents risk factors and *a*_*k*_ represents the estimated causal effects. UVMR-based determines *a*_*k*_ from MR-estimated causal effects ${\hat{\alpha }}_{k}$ for each risk factor *X*_*k*_, requiring at least five genome-wide significant genetic instruments, assigning *a*_*k*_ = 0 for factors with a lower number of instruments or nonsignificant causal effects (Bonferroni-corrected *P* < 0.05). Conversely, MVMR-based determines *a*_*k*_ jointly across all risk factors, through a multivariate regression of the risk factor effect size matrix *B*_*e*_ ∈ *R*^*S*×*K*^ on the outcome effect sizes *β*_*o*_ (Burgess and Thompson 2015; Sanderson 2021). Additionally, we contrast PRiMeR's performance with its linear counterpart, PRiMeR-LIN, adopting a linear disease risk prediction function *f*. As performance benchmarks, we also included supervised models trained directly on individual-level follow-up labels. We primarily compared PRiMeR against a LRM using the same risk prediction function as PRiMeR. Additionally, we conducted comparisons with ElasticNet, RandomForest, and XGBoost models. Hyperparameters for all models with access to individual-level follow-up labels were optimized using an inner fivefold cross-validation procedure (Supplemental Information). In simulation scenarios, these models aimed to minimize the mean squared error, whereas in the T2D study, the objective was to minimize the binary cross-entropy loss.

### Simulations

#### Data set generation

In our simulation study, we used 26 blood traits from 309,865 unrelated Europeans from the UKB data set, as potential risk factors. The data set is available at https://biobank.ndph.ox.ac.uk/ukb/ after a registration and approval process. We crafted scenarios where a subset of these traits exerted a causal influence on the health outcome, with individual risk contributions following a nonlinear increasing warping function—initially remaining negligible until surpassing a certain threshold, beyond which they increased linearly. The health outcome was generated as the sum of a linear combination of these nonlinearly transformed risk factors, a horizontal pleiotropy effect, and Gaussian noise. The horizontal pleiotropy effect was simulated as a direct genetic contribution from a subset of the variants associated with the blood traits. We systematically varied key parameters, such as the number of causal risk factors, and the proportions of the outcome variance explained by the risk factors and the horizontal pleiotropy effect, respectively. Additionally, we created a scenario in which we controlled the sharpness of the risk function, and whether it saturates after surpassing a certain threshold, instead of growing linearly. We jointly validated different values of sharpness and saturation, ranging from very flat risks to very abrupt risk increases, and from fully unbounded risks to those asymptotically approaching an upper bound. Finally, to assess the robustness of our model under conditions of limited genetic data, we explored scenarios with fewer genetic variants by randomly subsampling from the full set of variants associated with blood traits. For each simulation parameter configuration, we considered 10 repeat experiments, utilizing distinct random seeds. Detailed descriptions of our simulation approach can be found in Supplemental Information.

#### Evaluation framework

Adhering to a two-sample framework, the data were divided evenly into risk factor and outcome cohorts, with this consistent split maintained throughout all simulated scenarios. Through the genetic variant selection procedure detailed above, 2904 independent genetic variants were identified in the risk factor cohort. Using the outcome cohort, the effects of these genetic variants on the outcome were estimated—this step substitutes the real data analysis process of obtaining genetic effects on the outcome from external GWAS results. We trained PRiMeR using 80% of the risk factor cohort and evaluated the risk prediction accuracy on the remaining 20%. Prediction accuracy was assessed by calculating Spearman's correlation coefficient between the predicted and simulated risk values. Within this evaluation framework, we compare PRiMeR against PRiMeR-LIN, as well as UVMR-based and MVMR-based predictors. We also compared PRiMeR with models trained on individual-level follow-up data, including the LRM, ElasticNet, RandomForest, and XGBoost models (Supplemental Fig. A4). For these models, we considered inner fivefold cross-validation for hyperparameter selection (grid of explored values in Supplemental Information). Standard errors for all metrics were calculated from the results of 10 repeat experiments. To ensure the calibration of the evaluation procedure, Spearman's correlation coefficients of all MR-based models were verified to be compatible with zero in simulations without causal links (Supplemental Fig. A1).

### Diabetes risk predictions

#### Cohort definition

We utilized PRiMeR to predict 5-year T2D risk from 37 established risk factors (Edlitz and Segal 2022). For the outcome genetic effects and standard errors, we considered the external T2D GWAS summary statistics from Mahajan et al. (2018), which excluded the UKB cohort and can be obtained from http://www.type2diabetesgenetics.org/. For the risk factor cohort, we considered 218,665 unrelated Europeans from UKB who did not have diabetes at the time of assessment. After matching variants across the two data sets and excluding palindromic variants, the genetic variant selection procedure described above identified 6077 independent genetic variants associated with at least one of the 37 traits. More info on the longitudinal cohort definition can be found in Supplemental Information.

#### Evaluation framework

We compared PRiMeR with MR-based models (PRiMeR-LIN, UVMR-based, and MVMR-based) and models with direct access to individual-level follow-up data (LRM, ElasticNet, RandomForest, and XGBoost) (Supplemental Information). Additionally, we included a PRS predictor, computed externally by Thompson et al. (2024) and made available in UKB through field 26285, for comparison. To assess T2D risk prediction accuracy, we employed the AUC, using actual 5-year T2D risk as labels (derived from fields 41280 and 41270 using ICD10 code E11). To ensure robust significance testing and estimate standard errors, we conducted 50 repeat experiments with random 80%/20% splits for training and testing. Standard errors for all metrics were computed across these experiments, along with *t*-tests to assess performance improvements.

#### Interpretation of the learned T2D biomarker

To assess the risk predictor learned by PRiMeR in the T2D experiments, we employed analysis. Firstly, univariate associations were quantified using Spearman's correlation coefficients between the risk predictor and each input risk factor in out-of-sample individuals (Supplemental Fig. A6). Secondly, to evaluate the model's ability to capture nonlinear relationships among the selected risk factors, we visualized the learned contributions (normalized between 0 and 1) against observed values of these factors (Fig. 4C). We observed that all reported values are highly consistent across all 50 repeat experiments.

### Imaging biomarkers of dementia

#### Cohort definition

We employed two-sample MR methods to estimate the 5-year AD risk based on brain T1 MRI features. For the risk factor cohort, we selected 31,552 unrelated Europeans from UKB with T1 brain imaging data. Out of the 153 brain volume features that are available in UKB, we included 70 brain T1 MRI traits with at least five associated variants *P* < 5 × 10^−8^ as risk factors in all MR models, for which we identified a total of 385 genetic variants. More info on the risk factor cohort can be found in Supplemental Information. For outcome genetic effects, we utilized external GWAS summary stats for AD in unrelated Europeans from Wightman et al. (2021), which we downloaded from https://ctg.cncr.nl/software/summary_statistics.

#### Evaluation

We compared PRiMeR with PRiMeR-LIN, as well as UVMR-based and MVMR-based predictors. We assessed AD risk prediction accuracy by AUC using actual 5-year AD risk as labels (derived from field 131036). Across 31,552 unrelated Europeans from UKB with T1 brain imaging data, only 19 developed AD within 5 years. All models were trained on 80% of the healthy risk factor cohort, while the remaining 20% along with 19 individuals with reported AD were used as a test set. To robustly test for significance and estimate standard errors, we conducted 50 repeat experiments, each employing different random 80%/20% splits.

#### Interpretation of the learned AD biomarker

For the interpretation of the learned risk predictor, we conducted a voxel-based association analysis using T1-weighted MRI scans that were registered to the MNI152 template (Grabner et al. 2006; Miller et al. 2016; Alfaro-Almagro et al. 2018; https://www.bic.mni.mcgill.ca/ServicesAtlases/ICBM152NLin6). Each voxel's intensity was regressed against the out-of-sample individual's risk predictor scores, adjusting for sex, age, UKB array type, and the top 20 genetic principal components. This linear association test yielded *P*-values for the contribution of the risk predictor to each voxel, which were transformed into a heatmap overlay on the MNI152 template using signed *P*-values ( − log_10_(*P*) · sign(*β*)). Blue regions on the heat map indicate areas of volume decrease associated with increased risk predictor values, highlighting potential areas that correlate with higher AD risk (Fig. 4B). Furthermore, we quantified Spearman's correlation coefficient between the risk predictor and each input imaging trait within the held-out validation set (Fig. 4C; Supplemental Fig. A7). Overall, both analyses displayed remarkable consistency across all experimental repeats, underscoring the reliability of our findings.

### Accelerometer-based biomarkers for Parkinson's disease risk prediction

#### Cohort definition

We used PRiMeR to predict 5-year PD risk from accelerometer-derived features from Schalkamp et al. (2023). For outcome genetic effects and standard errors, we used external PD GWAS summary statistics from the FinnGen cohort, which is available at https://www.finngen.fi/en/access_results (Freeze 11; G6_PARKINSON). Our risk factor cohort included 69,670 from UKB without PD at the time of accelerometer data collection (field 90003). After matching variants across data sets and removing palindromic variants, we identified 45 independent variants associated (*P* < 5 × 10^−8^) with at least one of the 38 accelerometer traits, which were used as input features for our predictors. Due to the lower number of instruments, we repeated the experiment with a relaxed significance threshold for the inclusion of variants in genetics-based predictive modeling (*P* < 10^−6^), which yielded 185 variants and confirmed the robustness of our results across both thresholds (Supplemental Fig. A10). The Supplemental Information provides more details on the longitudinal cohort definition and the exact names of the considered features.

#### Evaluation framework

We compared the performance of PRiMeR against PRiMeR-LIN, as well as UVMR-based and MVMR-based predictors. Additionally, we included a PRS predictor, computed externally by Thompson et al. (2024) and made available in UKB through field 26260, for comparison. We assessed PD risk predictions, using the area under the receiver operating characteristic curve (AUC ROC) as our primary metric, based on actual 5-year PD risk labels (field 131022). Our cohort comprised 69,670 unrelated Europeans from the UKB with accelerometer data, of whom 128 developed PD within 5 years. We trained all models on 80% of healthy participants from the risk factor cohort and tested on the remaining 20%, along with the 128 PD cases. To ensure the robustness of our results, we show the standard error across 50 random 80%/20% splits.

#### Assessment of risk contribution

We computed Spearman's correlation coefficients between the predicted risk and the input accelerometer features within the test set (Supplemental Fig. A9). The values remained highly consistent across all 50 repeat experiments, highlighting the robustness of the results.

### Use of artificial intelligence

In the preparation of this manuscript, we utilized the large language model GPT-4 (https://chat.openai.com/) for editing assistance, including language polishing and clarification of text. Although this tool assisted in refining the manuscript's language, it was not used to generate contributions to the original research, data analysis, or interpretation of results. All final content decisions and responsibilities rest with the authors.

### Software availability

An open-source software implementation of PRiMeR and all baseline methods are available at GitHub (https://github.com/AIH-SGML/PRiMeR), Zenodo (https://doi.org/10.5281/zenodo.13632773), and as Supplemental Code.

## Supplementary Material

Supplement 1

Supplement 2
