# Supplementary material for: Genetics-driven risk predictions leveraging the Mendelian randomization framework
Source: Genome Res. 2024 Sep;34(9):1276–85. doi: 10.1101/gr.279252.124 (PMC11529896; doi:10.1101/gr.279252.124)
Supplement: Supplement 2 [file Supplemental_Material.pdf]

Supplementary Materials for "Genetics-driven Risk Predictions leveraging the Mendelian Randomization framework"

Contents

A1 Supplementary Information 1
A1.1 Related methods 1
A1.2 Detailed Description of PRiMeR 2
A1.3 Reduction to Multivariable MR for linear function 3
A1.4 Bayesian Extension 4
A1.5 Standard ML model baselines 4
A1.6 Simulations design 5
A1.7 Definition of the cohort for T2D onset prediction 6
A1.8 Definition of the cohort for Alzheimer's Dementia prediction 8
A1.9 Definition of the cohort for Parkinson's Disease prediction 9

A2 Supplementary Figures 11

A1 Supplementary Information

A1.1 Related methods

Risk predictions using longitudinal data. Risk prediction from electronic health records traditionally employs longitudinal data [15, 36]. Predictive models used for this task range from simple methods combining questionnaire responses [26, 33] to ML supervised models integrating multiple data types [4]. To model event timing and censored data, survival prediction methods like Cox regression become essential [9, 17, 38]. In contrast to approaches using longitudinal data, PRiMeR uses genetic effects on health outcomes as the supervisory signal to train risk predictors.

MR methods. The traditional MR methodology has seen numerous extensions tailored for varied settings. These encompass methods designed to address biases from weak instruments [46, 41, 45, 7, 42] and horizontal pleiotropy [8, 6, 22, 32, 47, 13, 40, 32, 46, 41, 29], as well as nonlinear models [10, 39, 37]. Among all these methods, PRiMeR is most related to multivariable MR [34, 11], which performs concurrent analyses of multiple exposures under linear assumptions, and nonlinear MR [39, 10], which enables the investigation of how a single exposure's causal effect varies for different values of the exposure [39, 10]. Importantly, PRiMeR does not aim to either discover or quantify a causal effect, but rather leverages the MR framework to learn a model for disease risk prediction.

Machine Learning in Instrument Variable Analysis. In the broader class of instrument variable analysis methods, the integration of machine learning (ML) techniques has recently begun to gain traction. For example, DeepIV leverages deep learning to predict treatment from high-dimensional instruments or intricate instrument structures [21, 3, 19]. In the realm of MR, Malina et al [28] combined MR with In silico mutagenesis to examine causal relationships between genomic marks and outcomes. Distinctly from these approaches, PRiMeR leverages gradient based optimization to learn nonlinear functions of multiple single-risk factors utilizing the MR framework.

Polygenic Risk Scores. Historically, predicting disease risk using genetic data has been realized through polygenic risk scores (PRSs), which estimate individuals' genetic risk by aggregating effects from associated genetic variants. This is commonly achieved through a GWAS, followed by procedures accounting for linkage disequilibrium to select independent loci [18, 31]. Notably, PRS can offer substantial potential for risk assessment and personalized healthcare [25, 5, 2], especially when integrated with risk trait data [25, 1, 23, 20, 24]. Unlike PRSs, which predict disease risk directly from genetic variants, PRiMeR only uses genetic data to learn a risk prediction model as a function of multiple single-risk factors.

A1.2 Detailed Description of PRiMeR

For a risk factor cohort consisting of N individuals, K risk factors, C covariates, and S independent genetic variants associated with at least one of the K risk factors, we define the following matrices and vectors:

- $\boldsymbol{X}$ represents the $N \times K$ matrix of risk factors;
- $\boldsymbol{F}$ represents the $N \times C$ matrix of covariates;
- $\boldsymbol{G}$ denotes the $N \times S$ matrix of genetic variants;
- $\boldsymbol{\beta}_o$ is the $S \times 1$ vector of genetic effects on the disease outcome, obtained from external GWAS summary statistics;
- $\boldsymbol{s}_o$ is the $S \times 1$ vector of standard errors associated with $\boldsymbol{\beta}_o$, also obtained from external GWAS summary statistics.

Within the PRiMeR framework, a differentiable function $f_{\boldsymbol{\phi}}$, parameterized by $\boldsymbol{\phi}$, transforms the input risk factors $\boldsymbol{X}$ into a single $N \times 1$ aggregate risk factor vector $\boldsymbol{e}(\boldsymbol{\phi}) = f_{\boldsymbol{\phi}}(\boldsymbol{X})$.* The PRiMeR framework treats the aggregate risk factor $\boldsymbol{e}(\boldsymbol{\phi})$ as the

*This operation is applied row-wise to each individual in $\boldsymbol{X}$.

exposure in an IVW regression model, formulated as:

$$\boldsymbol{\beta}_o \sim \mathcal{N}\left(\boldsymbol{\beta}_e(\boldsymbol{\phi})\alpha, \sigma^2 \,\mathrm{diag}(\boldsymbol{s}_o^2)\right). \tag{A.1}$$

Here, $\boldsymbol{\beta}_e(\boldsymbol{\phi})$ represents the marginal regression weights for each genetic variant in $\boldsymbol{G}$ against $\boldsymbol{e}(\boldsymbol{\phi})$, adjusting for covariates $\boldsymbol{F}$. For a single $N \times 1$ genetic variant $\boldsymbol{g}$, the regression model to estimate its effect (β_e) on a risk factor $\boldsymbol{e}(\boldsymbol{\phi})$ can be written as:

$$\boldsymbol{e}(\boldsymbol{\phi}) = \boldsymbol{g}\beta_e + \boldsymbol{F}\boldsymbol{\gamma} + \epsilon, \;\; \epsilon \sim \mathcal{N}\left(0, \sigma\,\mathbf{I}_N\right), \tag{A.2}$$

where $\mathbf{I}_N$ is the $N \times N$ identity matrix. Thus the maximum likelihood estimator (MLE) of $\beta_e(\boldsymbol{\phi})$ and $\boldsymbol{\gamma}(\boldsymbol{\phi})$ can be computed as:

$$\begin{bmatrix}\beta_e(\boldsymbol{\phi})\\ \boldsymbol{\gamma}(\boldsymbol{\phi})\end{bmatrix} = \left(\begin{bmatrix}\boldsymbol{g}^T\\ \boldsymbol{F}^T\end{bmatrix}\begin{bmatrix}\boldsymbol{g} & \boldsymbol{F}\end{bmatrix}\right)^{-1}\begin{bmatrix}\boldsymbol{g}\\ \boldsymbol{F}\end{bmatrix}\boldsymbol{e}(\boldsymbol{\phi}).,\tag{A.3}$$

from which we can derive the MLE of the genetic effect as a function $\boldsymbol{\phi}$ using block matrix inverse properties:

$$\beta_e(\boldsymbol{\phi}) = \underbrace{\left(\boldsymbol{g}^T\boldsymbol{g} - \boldsymbol{g}^T\boldsymbol{F}(\boldsymbol{F}^T\boldsymbol{F})^{-1}\boldsymbol{F}^T\boldsymbol{g}\right)^{-1}\boldsymbol{g}^T}_{\mathcal{M}(\boldsymbol{g},\boldsymbol{F})}\boldsymbol{e}(\boldsymbol{\phi}).\tag{A.4}$$

Thus, across all genetic variants, we have:

$$\boldsymbol{\beta}_e(\boldsymbol{\phi}) = h(\boldsymbol{e}(\boldsymbol{\phi}), \boldsymbol{G}, \boldsymbol{F}) = \begin{bmatrix}\mathcal{M}(\boldsymbol{G}_{:,1}, \boldsymbol{F})\boldsymbol{e}(\boldsymbol{\phi})\\ \cdots\\ \mathcal{M}(\boldsymbol{G}_{:,S}, \boldsymbol{F})\boldsymbol{e}(\boldsymbol{\phi})\end{bmatrix},\tag{A.5}$$

where $\boldsymbol{G}_{:,s}$ denotes the s-th column of $\boldsymbol{G}$, corresponding to the genotype vector of genetic variant s. For the from of the functional of $f_{\boldsymbol{\phi}}$, we adopt:

$$f_{\boldsymbol{\phi}}(\boldsymbol{x}) = \sum_{k=1}^{K} a_k \times \mathrm{ELU}(b_k x_k + c_k),\tag{A.6}$$

where the parameters $\boldsymbol{\phi} = \{a_1, \ldots, a_K, b_1, \ldots, b_K, c_1, \ldots, c_K\}$, and $\mathrm{ELU}(\cdot)$ is the Exponential Linear Unit activation function.

A1.3 Reduction to Multivariable MR for linear function

When adopting a linear function, PRiMeR reduces to the probabilistic model used in Multivariable MR. Indeed, when $e(\boldsymbol{\phi}) = \boldsymbol{X}\boldsymbol{\phi}$, Eq (A.5) becomes

$$\boldsymbol{\beta}_e(\boldsymbol{\phi}) = \begin{bmatrix}\mathcal{M}(\boldsymbol{G}_{:,1}, \boldsymbol{F})\boldsymbol{X}\boldsymbol{\phi}\\ \cdots\\ \mathcal{M}(\boldsymbol{G}_{:,S}, \boldsymbol{F})\boldsymbol{X}\boldsymbol{\phi}\end{bmatrix} = \underbrace{\begin{bmatrix}\mathcal{M}(\boldsymbol{G}_{:,1}, \boldsymbol{F})\boldsymbol{X}\\ \cdots\\ \mathcal{M}(\boldsymbol{G}_{:,S}, \boldsymbol{F})\boldsymbol{X}\end{bmatrix}}_{\boldsymbol{B}_e}\boldsymbol{\phi},\tag{A.7}$$

where $\boldsymbol{B}_e$ are the $S \times K$ marginal effects of the S genetic variants on the K risk factors. Replacing in Eq (A.10) we have

$$\boldsymbol{\beta}_o \sim \mathcal{N}\left(\boldsymbol{B}_e \underbrace{\boldsymbol{\phi}\alpha}_{\boldsymbol{\alpha}}, \sigma^2\mathrm{diag}(\boldsymbol{s}_o^2)\right),\tag{A.8}$$

where we redefined the effect size of the aggregate risk factor on the disease outcome as $\boldsymbol{\alpha}$. This is equivalent to the multivariable MR model introduced in [34, 11].

A1.4 Bayesian Extension

First, we note that by replacing the parametric form of f from Eq (A.6) in Eq (A.5), we have

$$\boldsymbol{\beta}_e(\boldsymbol{\phi}) = \underbrace{\begin{bmatrix} \mathcal{M}(\boldsymbol{G}_{:,1}, \boldsymbol{F})\tilde{\boldsymbol{X}}(\boldsymbol{b}, \boldsymbol{c}) \\ \dots \\ \mathcal{M}(\boldsymbol{G}_{:,S}, \boldsymbol{F})\tilde{\boldsymbol{X}}(\boldsymbol{b}, \boldsymbol{c}) \end{bmatrix}}_{\boldsymbol{B}_e(\boldsymbol{b},\boldsymbol{c})} \boldsymbol{a}, \tag{A.9}$$

where $\boldsymbol{\phi} = \{\boldsymbol{a}, \boldsymbol{b}, \boldsymbol{c}\}$, and we introduced transformed risk factors $\tilde{\boldsymbol{X}}(\boldsymbol{b}, \boldsymbol{c})$ through the ELU function, and the $S \times K$ marginal effects $\boldsymbol{B}_e(\boldsymbol{b}, \boldsymbol{c})$ of the S genetic variants on these K transformed risk factors. Replacing this expression into the IVW model in Eq (A.10), we have:

$$\boldsymbol{\beta}_o \sim \mathcal{N}\left(\boldsymbol{B}_e(\boldsymbol{b}, \boldsymbol{c})\, \boldsymbol{a}, \sigma^2 \mathrm{diag}(\boldsymbol{s}_o^2)\right). \tag{A.10}$$

where we absorbed parameter α into $\boldsymbol{a}$. Next, we extend this model to a Bayesian setting to enhance robustness when the number of genetic variants S is limited. Specifically, we introduce priors over the model parameters $\boldsymbol{a}$, $\boldsymbol{b}$ and $\boldsymbol{c}$:

$$\boldsymbol{a} \sim \mathcal{N}(\boldsymbol{0}, \sigma_a^2 \boldsymbol{I}), \tag{A.11}$$

$$\boldsymbol{b} \sim \mathcal{N}(\boldsymbol{0}, \sigma_b^2 \boldsymbol{I}), \tag{A.12}$$

$$\boldsymbol{c} \sim \mathcal{N}(\boldsymbol{0}, \sigma_c^2 \boldsymbol{I}), \tag{A.13}$$

where σ_a^2, σ_b^2 and σ_c^2 are the variances of the priors. The log marginal likelihood of the model, integrating out the parameters, is given by:

$$\begin{aligned} p(\boldsymbol{\beta}_o|\sigma_a^2, \sigma_b^2, \sigma_c^2) &= \int \log \mathcal{N}\left(\boldsymbol{\beta}_o|\boldsymbol{B}_e(\boldsymbol{b}, \boldsymbol{c})\boldsymbol{a}, \sigma^2 \mathrm{diag}(\boldsymbol{s}_o^2)\right) \\ &\quad \times \mathcal{N}(\boldsymbol{a}|\boldsymbol{0}, \sigma_a^2\boldsymbol{I})\mathcal{N}(\boldsymbol{b}|\boldsymbol{0}, \sigma_b^2\boldsymbol{I})\mathcal{N}(\boldsymbol{c}|\boldsymbol{0}, \sigma_c^2\boldsymbol{I})\, d\boldsymbol{a}\, d\boldsymbol{b}\, d\boldsymbol{c} \\ &= \int \underbrace{\log \mathcal{N}\left(\boldsymbol{\beta}_o|\boldsymbol{0}, \sigma_a^2 \boldsymbol{B}_e(\boldsymbol{b}, \boldsymbol{c})\boldsymbol{B}_e(\boldsymbol{b}, \boldsymbol{c})^T + \sigma^2 \mathrm{diag}(\boldsymbol{s}_o^2)\right)}_{\log p(\boldsymbol{\beta}_o|\boldsymbol{b},\boldsymbol{c},\sigma_a^2)} \\ &\quad \times \mathcal{N}(\boldsymbol{b}|\boldsymbol{0}, \sigma_b^2\boldsymbol{I})\mathcal{N}(\boldsymbol{c}|\boldsymbol{0}, \sigma_c^2\boldsymbol{I})\, d\boldsymbol{b}\, d\boldsymbol{c}, \end{aligned} \tag{A.14}$$

where in the last passage we analytically integrated over $\boldsymbol{a}$. As the remaining integral is intractable, we employ mean-field variational inference to derive an Evidence Lower Bound (ELBO):

$$\begin{aligned} \mathrm{ELBO}(\sigma_a^2, \sigma_b^2, \sigma_c^2, \boldsymbol{\lambda}) &= \mathbb{E}_{q_{\boldsymbol{\lambda}}(\boldsymbol{b},\boldsymbol{c})}[\log p(\boldsymbol{\beta}_o|\boldsymbol{b}, \boldsymbol{c}, \sigma_a^2)] \\ &\quad - \mathrm{KL}[q_{\boldsymbol{\lambda}}(\boldsymbol{b}, \boldsymbol{c})||\mathcal{N}(\boldsymbol{b}|\boldsymbol{0}, \sigma_b^2\boldsymbol{I})\mathcal{N}(\boldsymbol{c}|\boldsymbol{0}, \sigma_c^2\boldsymbol{I})], \end{aligned} \tag{A.15}$$

where $q_{\boldsymbol{\lambda}}(\boldsymbol{b}, \boldsymbol{c})$ is the family of fully factorized Gaussian distributions on $\boldsymbol{b}$ and $\boldsymbol{c}$ parameterized by variational parameters $\boldsymbol{\lambda} = \{\boldsymbol{b}_m, \boldsymbol{b}_s, \boldsymbol{c}_m, \boldsymbol{c}_s\}$, and KL denotes the Kullback-Leibler divergence. The ELBO is maximized with respect to σ_a^2 and $\boldsymbol{\lambda}$ using gradient ascent, employing the reparameterization Trick for gradients through the expectation term. Prior parameters σ_b^2 are σ_c^2 are set to 0.8 and 3 respectively, based on our prior assumptions on the type of nonlinearities we will see in the data.

A1.5 Standard ML model baselines

In this section, we elaborate on the training procedure and implementation details of the supervised machine learning models that have access to individual-level follow-up labels: ElasticNet (EN), RandomForest (RF), and XGBoost (XGB).

Training Procedure. Hyperparameter optimization was conducted for all models using a grid search approach. For each combination of parameters within the grid, an inner 5-fold cross-validation was performed. The performance of each hyperparameter configuration was assessed based on the average metric across all folds. After identifying the optimal hyperparameters, the models were refitted on the entire training dataset and subsequently evaluated on the same held-out validation set of PRiMeR. In the simulations, the mean squared error served as the performance metric for hyperparameter tuning, while in the type 2 diabetes (T2D) experiments, binary cross-entropy was used for the RandomForest and XGBoost models.

Implementation Details. The ElasticNet and RandomForest models were implemented using the regressor and classifier classes, respectively, from the `scikit-learn` [30] Python package for simulations and T2D experiments. For XGBoost, we employed the respective regressor and classifier implementations from the `XGBoost` Python package [12]. The following hyperparameter grids were utilized for optimization across both simulations and T2D experiments:

ElasticNet. The hyperparameter grid for ElasticNet was as follows:

- `l1_ratio`: [0.1, 0.5, 0.7, 0.9, 0.95, 0.99, 1.0]
- `eps`: 5.0e-4
- `n_alphas`: 500
- `max_iter`: 5000

RandomForest. The hyperparameter grid for RandomForest included:

- `n_estimators`: [100, 200, 300]
- `max_depth`: [10, 20, 30, None]
- `min_samples_split`: [2, 5, 10]
- `min_samples_leaf`: [1, 2, 4]

XGBoost. The hyperparameter grid for XGBoost was composed of:

- `n_estimators`: [100, 200, 300]
- `max_depth`: [3, 9, 0]
- `learning_rate`: [0.01, 0.05, 0.1]
- `subsample`: [0.6, 0.8, 1.0]
- `colsample_bytree`: [0.6, 0.8, 1.0]

A1.6 Simulations design

For N individuals and S independent genetic variants, associated with at least one of K risk factors, let $\boldsymbol{G}$ denote the $N \times S$ genotype matrix and $\boldsymbol{S}$ denote the $N \times K$ risk factor matrix. The outcome is simulated as the sum of three contributions:

$$\boldsymbol{y} = \underbrace{\boldsymbol{e}_{\text{sim}}}_{\text{simulated risk}} + \underbrace{\boldsymbol{h}_{\text{pleio}}}_{\text{horizontal pleiotropy}} + \underbrace{\boldsymbol{\epsilon}}_{\text{Gaussian noise}} \tag{A.16}$$

Given the number of risk factors with a directional effect (K_c), the variance explained by simulated risk (v_{caus}), and the variance explained by horizontal pleiotropy (v_{pleio}), each term was simulated as described in the following:

1. **Simulated risk** ($\boldsymbol{e}_{\text{sim}}$)
   - Select K_c single-risk factors with indices $\{i_1, \ldots, i_{K_c}\}$
   - Simulate risk

   $$\boldsymbol{e}_{\text{sim}} = \frac{1}{K_c} * \sum_{k}^{K_c} \text{std}(u(\boldsymbol{E}_{:,i_k} - \text{quantile}(\boldsymbol{E}_{:,i_k}, 0.75))) \tag{A.17}$$

   where

   $$u(\boldsymbol{x}) = (1 - \lambda) \cdot \text{ELU}(\gamma \cdot \boldsymbol{x}) + \lambda \cdot \hat{\sigma}(\gamma \cdot \boldsymbol{x}), \tag{A.18}$$

   where ELU denotes the exponential linear unit function, and $\hat{\sigma}(x) = 2 * \sigma(2 \cdot x) - 1$ is a rescaled and shifted sigmoid function, sutch that the turning point at which the risk either increases linearly ($\lambda = 0$) or starts to saturate ($\lambda = 1$) is always at $x = 0$. We used $\gamma = 3.16$ and $\lambda = 0$ as default parameters, but also evaluated the robustness of our results across different choices in Figure A2. Moreover, std$(\cdot)$ denotes the standardization function across N individuals, which ensures that every contributing risk factor explains the same variance;
   - Rescale such that $\boldsymbol{e}_{\text{sim}}$ has variance v_{caus}

   $$\boldsymbol{e}_{\text{sim}} \leftarrow \sqrt{v_{\text{caus}}}\, \boldsymbol{e}_{\text{sim}} \tag{A.19}$$

2. **Horizontal Pleiotropy** ($\boldsymbol{h}_{\text{pleio}}$)
   - Select 10% genetic variants and standardize, $\boldsymbol{G}_c = \text{std}(\boldsymbol{G}[:, \{s_1, \ldots, s_H\}])$
   - Simulate genetic effects and standardize

   $$\boldsymbol{h}_{\text{pleio}} = \text{std}(\boldsymbol{G}_c \boldsymbol{b}), \quad \text{with } \boldsymbol{b} \overset{\text{iid}}{\sim} \text{Unif}(\{-1, 1\}) \tag{A.20}$$

   - Rescale such that $\boldsymbol{h}_{\text{pleio}}$ has variance v_{pleio}

   $$\boldsymbol{h}_{\text{pleio}} \leftarrow \sqrt{v_{\text{pleio}}}\, \boldsymbol{h}_{\text{pleio}} \tag{A.21}$$

3. **Gaussian noise** ($\boldsymbol{\epsilon}$)

   $$\boldsymbol{\epsilon} = \sqrt{1 - v_{\text{caus}} - v_{\text{pleio}}} \cdot \text{std}(\boldsymbol{\eta}), \quad \text{with } \boldsymbol{\eta} \overset{\text{iid}}{\sim} \mathcal{N}(0, 1) \tag{A.22}$$

A1.7 Definition of the cohort for T2D onset prediction

Cohort definition. To test the performance of PRiMeR for the prediction of T2D development, we retrospectively created a cohort from UKB (see Figure 4a). We considered unrelated Europeans from UKB who did not have diabetes at the time of assessment. This was ensured by filtering the population based on the following criteria:

- Do not have reports of any kind of diabetes before the baseline or within the first six months after the assessment. The latter guarantees that the diagnosis was not given directly after, based on the data collected at the baseline. The comparison is done using the following fields: 130706, 130708, 130710, 130712, 130714, 130716, 130718, and Instance 0.0 of field 53.
- Do not have a critical level of the main biomarker for T2D, HbA1c (HbA1c $<$ 48 mmol/mol) at the baseline (Instance 0.0, field 30750).
- Medication-based exclusion of patients from the initial cohort that reported taking one of the following drugs at the time of baseline assessment: 'metformin', 'glibenclamide', 'glimepiride', 'repaglinide', 'nateglinide', 'troglitazone', 'pioglitazone', 'rosiglitazone', 'rosiglitazone 1mg/metformin 500mg tablet', 'acarbose', 'gliclazide', 'glipizide product', 'tolbutamide', 'insulin product'. The data was taken from field 20003.

As we were aiming to predict the T2D development in 5 years after assessment, some additional censoring was also implemented. Patients who have died or did not have Health and Social Care Information Centre (HES) or General Practitioner (GP) records during the 5 years of follow-up would not have received the right T2D diagnosis if they had the disease. Hence, they were removed from the cohort.
After these exclusions, the size of the cohort was 218,665 people. According to the ICD10 medical records in fields 41270 and 41280, 1,490 patients in the cohort developed type 2 diabetes (code E11) within the next 5 years and were further considered as "cases". The rest of the filtered cohort was considered to be controls.

Risk factor selection. The prediction of T2D is based on the following 37 predictive factors:

- 26 blood biomarkers (4 were excluded because of high percentage of missing data):
  - Albumin (field 30600)
  - Alkaline Phosphatase (field 30610)
  - Alanine Aminotransferase (field 30620)
  - Apolipoprotein A (field 30630)
  - Apolipoprotein B (field 30640)
  - Aspartate Aminotransferase (field 30650)
  - Urea (field 30670)
  - Calcium (field 30680)
  - Cholesterol (field 30690)
  - Creatinine (field 30700)
  - C-Reactive Protein (field 30710)
  - Cystatin C (field 30720)
  - Gamma-Glutamyltransferase (field 30730)
  - Glucose (field 30740)
  - Glycated Hemoglobin (field 30750)
  - HDL Cholesterol (field 30760)

  - IGF-1 (field 30770)
  - LDL Direct (field 30780)
  - Phosphate (field 30810)
  - SHBG (field 30830)
  - Total Bilirubin (field 30840)
  - Testosterone (field 30850)
  - Total Protein (field 30860)
  - Triglycerides (field 30870)
  - Urate (field 30880)
  - Vitamin D (field 30890)
- Diastolic blood pressure, automated reading (field 4079)
- Systolic blood pressure, automated reading (field 4080)
- Pulse rate, automated reading (field 102)
- Waist-Hips circumference ratio, calculated from Waist circumference (field 48) and Hip circumference (field 49) is
- BMI, Body mass index (field 21001)
- 6 blood-count features (easiest to measure):
  - Hemoglobin concentration (field 30020)
  - Mean corpuscular volume (field 30040)
  - Mean platelet (thrombocyte) volume (field 30100)
  - Red blood cell (erythrocyte) count (field 30010)
  - White blood cell (leukocyte) count (field 30000)
  - Platelet count (field 30080)

Missing values within the data were imputed using the mean of the respective feature.

A1.8 Definition of the cohort for Alzheimer's Dementia prediction

To test the performance of PRiMeR for the prediction of dementia, we created a cohort from UKB with 31,552 unrelated individuals of European descent with available T1 MRI brain imaging data. As predictive features, we used brain T1 MRI volume measurements from categories 1101 and 1102 of UKB. Out of these 153 brain volume features, we selected 70 traits, which had at least 5 genetic variants ($P < 5 \cdot 10^{-8}$) identified by our variant selection procedure (**Methods**). Missing values within the data were imputed using the mean of the respective feature.

A1.9 Definition of the cohort for Parkinson's Disease prediction

To evaluate PRiMeRs performance in predicting Parkinson's Disease (PD) development, we considered a subset of the 103,712 individuals in UKB for which accelerometer data [43, 16, Category 1008] was available. Our final cohort comprised 67,199 unrelated Europeans without PD at baseline, of whom 124 developed PD within 5 years. PD cases were identified based on the date of diagnosis (field 131022), including participants without a PD diagnosis at baseline or whose PD diagnosis occurred within 5 years following the initial accelerometer measurement (field 90003).
Participants included in the study had accelerometer data (field 90003), passed quality control for accelerometer data (field 90016), and had sufficient wear time (field 90015). Individuals who died within 5 years post-accelerometer measurement (field 40000) were excluded to ensure adequate follow-up duration. Participants who reported using anti-parkinsonism drugs before or during the accelerometer wear period (up to 7 days post-initiation) were also excluded, based on medication data (field 20003), to avoid significant effects of medications on acceleration measurements. The list of anti-parkinsonism drugs was defined using the Anatomical Therapeutic Chemical (ATC) classification code N04 [44].
Following the procedures described by Schalkamp et al. [35], we computed 69 accelerometer derived features and conduct a GWAS for each feature, where missing values were imputed using the mean of the respective feature. Following our variant selection procedure (**Methods)** we obtained 45 independent genetic variants which are associated with at least one of the 69. After dropping features which did not have at least one associated genetic variant we arrive at the following 38 for the experiment:

- Average Acceleration from 1am onwards (1-hour period)
- Average Acceleration from 2am onwards (1-hour period)
- Average Acceleration from 3am onwards (1-hour period)
- Average Acceleration from 5am onwards (1-hour period)
- Average Acceleration from 7am onwards (1-hour period)
- Average Acceleration from 8am onwards (1-hour period)
- Average Acceleration from 11pm onwards (1-hour period)
- Average Acceleration from 12am onwards (1-hour period)
- Average Acceleration from 1pm onwards (1-hour period)
- Average Acceleration from 6pm onwards (1-hour period)
- Average Acceleration on Monday
- Average Acceleration on Friday
- Average Acceleration on Sunday
- Average Light Activity Duration per 24 hours
- Average Maximum Consecutive Light Activity Duration per 24 hours

- Average Maximum Consecutive Moderate-to-Vigorous Activity Duration per 24 hours
- Average Maximum Consecutive Sedentary Activity Duration per 24 hours
- Average Maximum Consecutive Sleep Duration per 24 hours
- Average Moderate-to-Vigorous Activity Duration per 24 hours
- Average Movement during Light Activity
- Average Movement during Sedentary Activity
- Average Movement during Sleep
- Average Number of Light Activity Intervals per 24 hours
- Average Number of Moderate-to-Vigorous Activity Intervals per 24 hours
- Average Number of Sedentary Intervals between 11pm and 7am
- Average Number of Sleep Intervals between 11pm and 7am
- Average Number of Sleep Intervals between 7am and 11pm (only used during relaxed run (**Methods**))
- Average Number of Sleep Intervals per 24 hours
- Average Sedentary Activity Duration per 24 hours
- Average Sleep Duration per 24 hours
- Maximum Consecutive Light Activity Duration
- Maximum Consecutive Sedentary Activity Duration
- Maximum Consecutive Sleep Duration
- Standard Deviation of Acceleration over the Week
- Standard Deviation of Moderate-to-Vigorous Activity Duration per 24 hours
- Standard Deviation of Movement during Sedentary Activity
- Standard Deviation of Movement during Sleep
- Standard Deviation of Sedentary Activity Duration per 24 hours

A2 Supplementary Figures

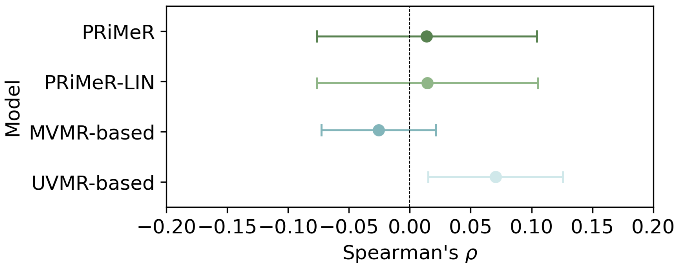

Figure A1: **Model calibration under simulations with no directional effects.** The figure presents Spearman's correlation coefficients (ρ) between simulated risk factors and predictions from PRiMeR, PRiMeR-LIN, MVMR-based, and UVMR-based on the held-out validation set, when no directional effects are simulated. Error bars denote standard errors across 10 repeat experiments. The dashed line at $\rho = 0$ indicates the expected random chance correlations in this simulated setting. These results confirm calibration of our simulation and evaluation procedure.

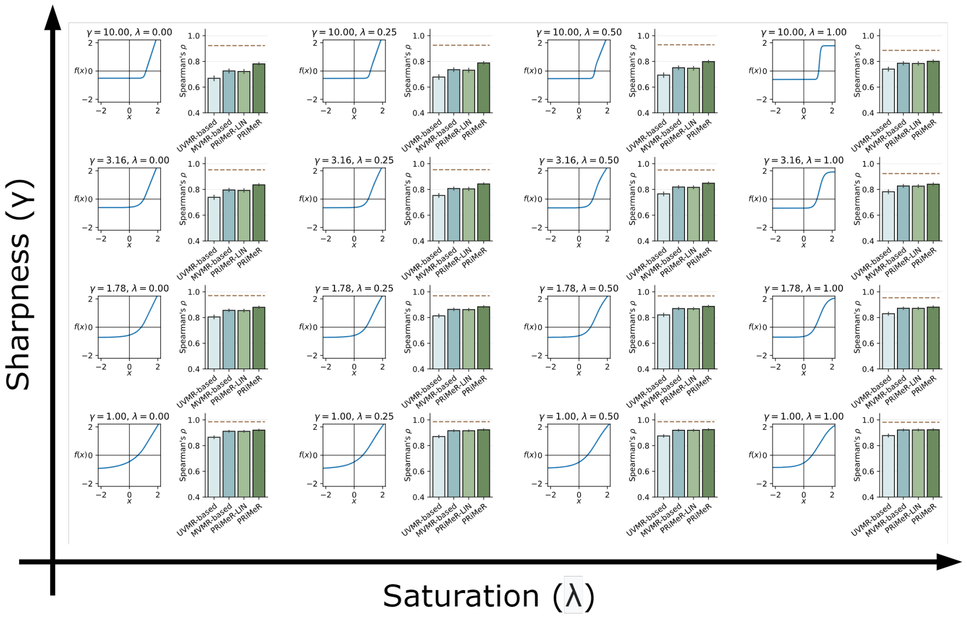

Figure A2: **Simulations of Different Single-Risk Factor Monotonic Functions.** Each subfigure displays a specific single-risk factor monotonic function used to simulate the aggregate risk (left panel) and the corresponding performance of all methods (right panel). The sharpness of the function varies from less sharp (bottom) to sharper (top), and saturation from non-saturating (left) to saturating (right), parameterized by γ and λ respectively. Refer to Section A1.6 and Eq (A.18) for details. Bar plots indicate the mean Spearman's correlation coefficients (ρ) between the simulated and aggregate risk factor, with standard error shown across 10 repeat experiments. These results show that PRiMeRs performance is robust across all settings, outperforming the comparison methods in scenarios where the risk increases abruptly and does not saturate.

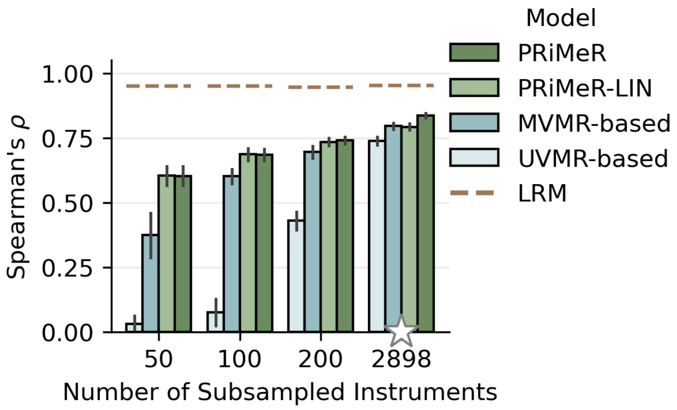

Figure A3: **Simulations with Varying Number of Genetic Variants.** This figure illustrates the mean Spearman's correlation coefficient (ρ) and standard error across 10 repeat experiments for different numbers of subsampled genetic variants. Details are provided in the Methods section. The results demonstrate that both PRiMeR and PRiMeR-LIN exhibit more robust performance in scenarios with limited genetic variants, compared to MVMR-based and UVMR-based predictors. This robustness stems from the Bayesian framework implemented in PRiMeR. Notably, PRiMeR-LIN and MVMR-based models share the same likelihood function; the distinguishing factor is the Bayesian prior on regression weights in PRiMeR-LIN, which provides regularization in scenarios with a low number of instruments.

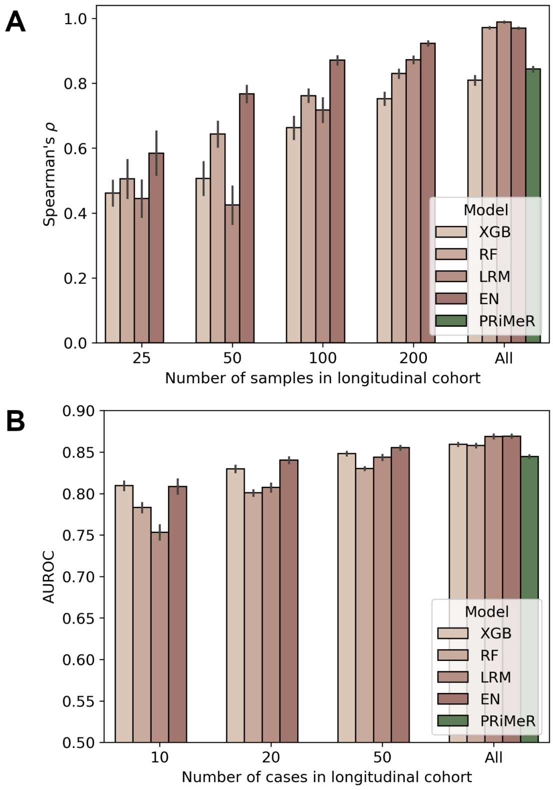

Figure A4: **Comparison with Supervised Longitudinal Models for Varying Cohort Size.** This figure demonstrates the predictive performance of various supervised machine learning models as the number of samples with available follow-up labels varies, compared with PRiMeR, which is trained on "all" data and does not rely on follow-up labels. Panel (**a**) showcases comparisons in simulations, using Spearman's correlation coefficient (ρ) between the predicted outcomes and simulated aggregate risks as the metric for prediction accuracy. To evaluate the performance of longitudinal models across different cohort sizes, we simulate training sets with varying sample sizes and evaluate all models on the same held-out test set. The models include ElasticNet (EN), RandomForest (RF), XGBoost (XGB), and a model with the same functional form as PRiMeR's risk prediction function (Longitudinal Reference Model; LRM). Panel (**b**) presents a similar analysis in a type 2 diabetes dataset, where the training subset is modified to contain a specific number of future cases while maintaining a fixed incidence rate, and the test set is consistent across all experiments. We use the area under the receiver operating characteristic curve (AUROC) for 5-year T2D risk predictions as the prediction accuracy metric, comparing the same set of models adapted for classification. These results illustrate the sample size of the longitudinal cohort needed to surpass the predictive power of PRiMeR.

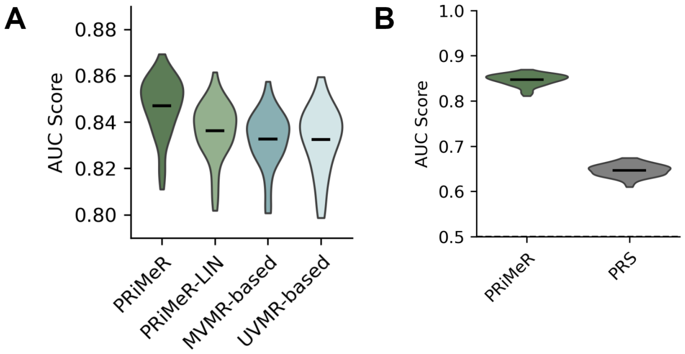

Figure A5: **Distributions of AUC Scores for Type 2 Diabetes Risk Prediction.** Panel (**a**) displays the distribution of AUC scores for PRiMeR, PRiMeR-LIN, MVMR-based, and UVMR-based models. Panel (**b**) contrasts the distribution of the AUC score of PRiMeR against a polygenic risk score (PRS) model. In both Panels the black horizontal bar indicates the mean value across 50 repeat experiments.

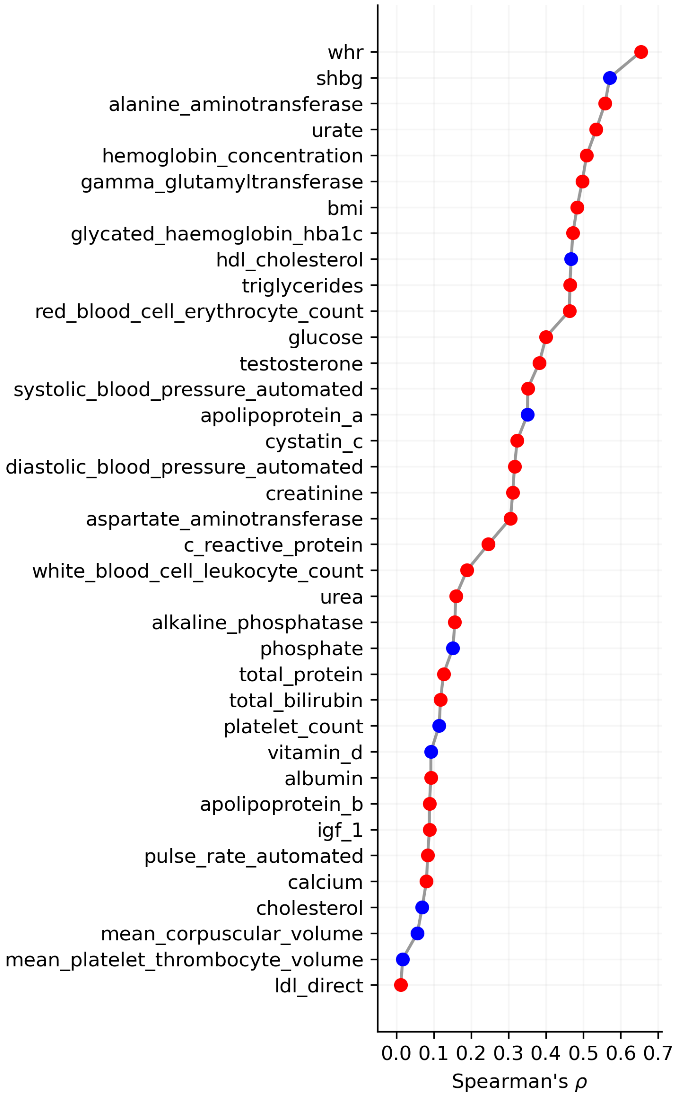

Figure A6: **Correlation of the T2D risk predictions with clinical blood markers.** Shown are the mean Spearman's correlation coefficients (ρ) between the aggregate risk factor of PRiMeR and the analyzed blood traits on the test set across 50 repeat experiments. Red dots represent markers with positive correlations, while blue dots indicate negative correlations. Shown are the average correlations on the test set of 50 repeat experiments.

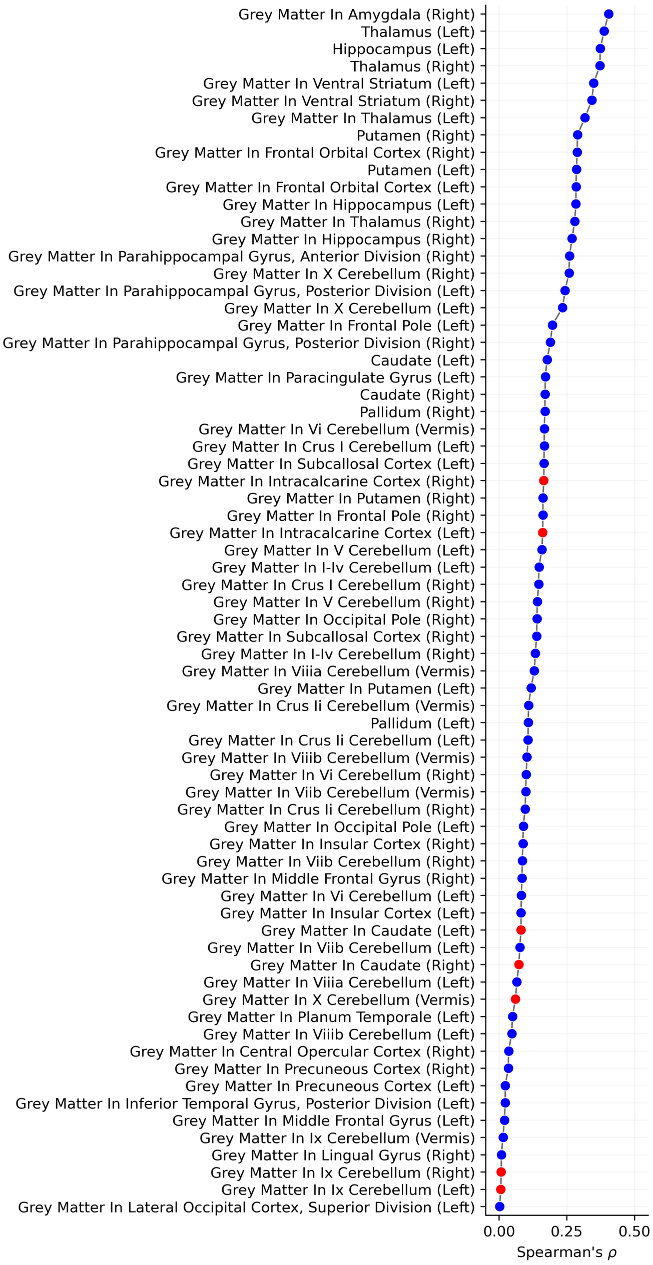

Figure A7: **Correlation of the AD risk predictions with T1 imaging traits.** Shown are the mean Spearman's correlation coefficients (ρ) between the aggregate risk of PRiMeR and the analyzed T1 MRI features on the test set across 50 repeat experiments. Red dots represent markers with positive correlations, while blue dots indicate negative correlations. The strongest negative correlations, indicative of gray matter reduction, are observed in regions known to be impacted by Alzheimer's pathology, such as the amygdala and hippocampus [14].

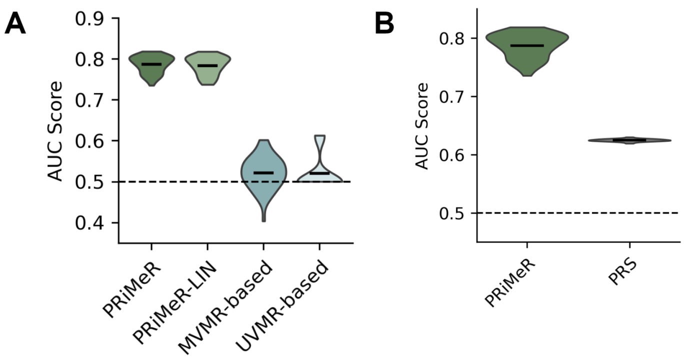

Figure A8: **Distributions of AUC Scores for Parkinson's Disease Risk Prediction.** Panel (**a**) displays the distribution of AUC scores for PRiMeR, PRiMeR-LIN, MVMR-based, and UVMR-based models. Panel (**b**) contrasts the distributions of PRiMeR against a polygenic risk score (PRS) model, which was evaluated using the same scheme as PRiMeR, which is detailed in our **Methods**. The horizontal black bars in both panels indicate the mean across 50 repeat experiments.

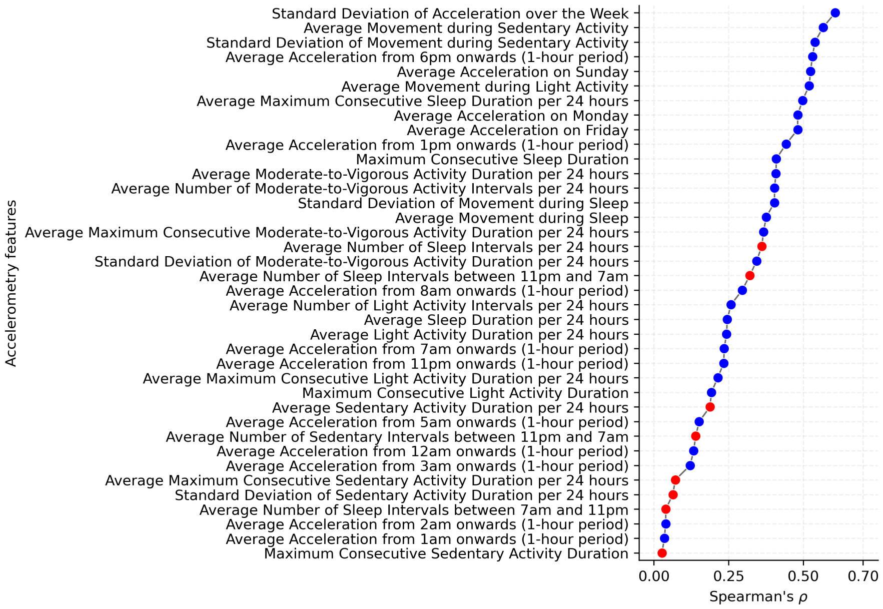

Figure A9: **Correlation of PD Risk Predictions with Accelerometer derived Features.** Shown are the mean Spearman's correlation coefficients (ρ) between the aggregate risk of PRiMeR and the considered accelerometer features on the test set and across 50 repeat experiments. Red dots represent features with positive correlations, while blue dots indicate negative correlations. Top features such as "Average Maximum Consecutive Sleep Duration per 24 hours" and "Maximum Consecutive Sleep Duration" emphasize the role of sleep quality and duration [27]. Furthermore, features like "Standard deviation of Average Acceleration over the Week" and "Average Movement during Sedentary Activity" reflect the importance of physical activity, as reduced mobility is commonly observed in PD patients. These results highlight the relevance of non-motor symptoms and physical activity monitoring in assessing PD risk [35].

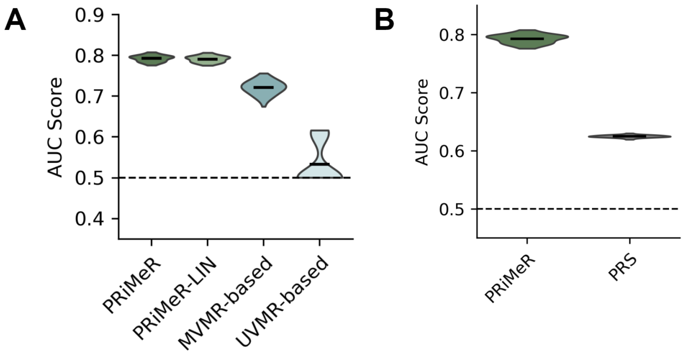

Figure A10: **AUC Scores for Parkinson's risk prediction using a relaxed threshold $P < 10^{-6}$ for instrument selection.** Panel (**a**) displays the distribution of AUC scores for PRiMeR, PRiMeR-LIN, MVMR-based, and UVMR-based models. Panel (**b**) contrasts the distributions of PRiMeR against a polygenic risk score (PRS) model, which was evaluated using the same scheme as PRiMeR, which is detailed in our **Methods**. The horizontal black bars in both panels indicate the mean across 50 repeat experiments.

References

[1] Abraham G, Havulinna AS, Bhalala OG, Byars SG, De Livera AM, Yetukuri L, Tikkanen E, Perola M, Schunkert H, Sijbrands EJ, et al.. 2016. Genomic prediction of coronary heart disease. European heart journal **37**: 3267–3278.

[2] Adeyemo A, Balaconis MK, Darnes DR, Fatumo S, Moreno PG, Hodonsky CJ, Inouye M, Kanai M, Kato K, Knoppers BM, et al.. 2021. Responsible use of polygenic risk scores in the clinic: potential benefits, risks and gaps. Nature Medicine **27**: 1876–1884.

[3] Bennett A, Kallus N, and Schnabel T. 2019. Deep generalized method of moments for instrumental variable analysis. Advances in neural information processing systems **32**.

[4] Bennis FC, Hoogendoorn M, Aussems C, and Korevaar JC. 2022. Prediction of heart failure 1 year before diagnosis in general practitioner patients using machine learning algorithms: a retrospective case–control study. BMJ open **12**: e060458.

[5] Bowden J, Davey Smith G, and Burgess S. 2015. Mendelian randomization with invalid instruments: effect estimation and bias detection through egger regression. International journal of epidemiology **44**: 512–525.

[6] Bowden J, Davey Smith G, Haycock PC, and Burgess S. 2016. Consistent estimation in mendelian randomization with some invalid instruments using a weighted median estimator. Genetic epidemiology **40**: 304–314.

[7] Bowden J, Del Greco M F, Minelli C, Zhao Q, Lawlor DA, Sheehan NA, Thompson J, and Davey Smith G. 2019. Improving the accuracy of two-sample summary-data mendelian randomization: moving beyond the nome assumption. International journal of epidemiology **48**: 728–742.

[8] Bowden J, Spiller W, Del Greco M F, Sheehan N, Thompson J, Minelli C, and Davey Smith G. 2018. Improving the visualization, interpretation and analysis of two-sample summary data mendelian randomization via the radial plot and radial regression. International journal of epidemiology **47**: 1264–1278.

[9] Bragg F, Trichia E, Aguilar-Ramirez D, Bešević J, Lewington S, and Emberson J. 2022. Predictive value of circulating nmr metabolic biomarkers for type 2 diabetes risk in the uk biobank study. BMC medicine **20**: 159.

[10] Burgess S, Davies NM, Thompson SG, Consortium EI, et al.. 2014. Instrumental variable analysis with a nonlinear exposure–outcome relationship. Epidemiology **25**: 877–885.

[11] Burgess S and Thompson SG. 2015. Multivariable Mendelian Randomization: The Use of Pleiotropic Genetic Variants to Estimate Causal Effects. American Journal of Epidemiology **181**: 251–260.

[12] Chen T and Guestrin C. 2016. XGBoost: A scalable tree boosting system. In Proceedings of the 22nd ACM SIGKDD International Conference on Knowledge Discovery and Data Mining, KDD '16, pp. 785–794. ACM, New York, NY, USA.

[13] Cho Y, Haycock PC, Sanderson E, Gaunt TR, Zheng J, Morris AP, Davey Smith G, and Hemani G. 2020. Exploiting horizontal pleiotropy to search for causal pathways within a mendelian randomization framework. Nature communications **11**: 1010.

[14] Coupé P, Manjón JV, Lanuza E, and Catheline G. 2019. Lifespan changes of the human brain in alzheimer's disease. Scientific Reports **9**: 3998.

[15] Darke P, Cassidy S, Catt M, Taylor R, Missier P, and Bacardit J. 2022. Curating a longitudinal research resource using linked primary care ehr data—a uk biobank case study. Journal of the American Medical Informatics Association **29**: 546–552.

[16] Doherty A, Jackson D, Hammerla N, Plötz T, Olivier P, Granat MH, White T, Van Hees VT, Trenell MI, Owen CG, et al.. 2017. Large scale population assessment of physical activity using wrist worn accelerometers: the uk biobank study. PloS one **12**: e0169649.

[17] Edlitz Y and Segal E. 2022. Prediction of type 2 diabetes mellitus onset using logistic regression-based scorecards. Elife **11**: e71862.

[18] Euesden J, Lewis CM, and O'reilly PF. 2015. Prsice: polygenic risk score software. Bioinformatics **31**: 1466–1468.

[19] Farrell MH, Liang T, and Misra S. 2021. Deep neural networks for estimation and inference. Econometrica **89**: 181–213.

[20] Hahn SJ, Kim S, Choi YS, Lee J, and Kang J. 2022. Prediction of type 2 diabetes using genome-wide polygenic risk score and metabolic profiles: A machine learning analysis of population-based 10-year prospective cohort study. EBioMedicine **86**.

[21] Hartford J, Lewis G, Leyton-Brown K, and Taddy M. 2017. Deep iv: A flexible approach for counterfactual prediction. In International Conference on Machine Learning, pp. 1414–1423. PMLR.

[22] Hartwig FP, Davey Smith G, and Bowden J. 2017. Robust inference in summary data mendelian randomization via the zero modal pleiotropy assumption. International journal of epidemiology **46**: 1985–1998.

[23] Inouye M, Abraham G, Nelson CP, Wood AM, Sweeting MJ, Dudbridge F, Lai FY, Kaptoge S, Brozynska M, Wang T, et al.. 2018. Genomic risk prediction of coronary artery disease in 480,000 adults: implications for primary prevention. Journal of the American College of Cardiology **72**: 1883–1893.

[24] Läll K, Mägi R, Morris A, Metspalu A, and Fischer K. 2017. Personalized risk prediction for type 2 diabetes: the potential of genetic risk scores. Genetics in Medicine **19**: 322–329.

[25] Lambert SA, Abraham G, and Inouye M. 2019. Towards clinical utility of polygenic risk scores. Human molecular genetics **28**: R133–R142.

[26] Lindstrom J and Tuomilehto J. 2003. The diabetes risk score: a practical tool to predict type 2 diabetes risk. Diabetes care **26**: 725–731.

[27] Lysen TS, Darweesh SKL, Ikram MK, Luik AI, and Ikram MA. 2019. Sleep and risk of parkinsonism and Parkinson's disease: a population-based study. Brain **142**: 2013–2022.

[28] Malina S, Cizin D, and Knowles DA. 2022. Deep mendelian randomization: Investigating the causal knowledge of genomic deep learning models. PLOS Computational Biology **18**: e1009880.

[29] Morrison J, Knoblauch N, Marcus JH, Stephens M, and He X. 2020. Mendelian randomization accounting for correlated and uncorrelated pleiotropic effects using genome-wide summary statistics. Nature genetics **52**: 740–747.

[30] Pedregosa F, Varoquaux G, Gramfort A, Michel V, Thirion B, Grisel O, Blondel M, Prettenhofer P, Weiss R, Dubourg V, et al.. 2011. Scikit-learn: Machine learning in Python. Journal of Machine Learning Research **12**: 2825–2830.

[31] Privé F, Vilhjálmsson BJ, Aschard H, and Blum MG. 2019. Making the most of clumping and thresholding for polygenic scores. The American Journal of Human Genetics **105**: 1213–1221.

[32] Rees JM, Wood AM, Dudbridge F, and Burgess S. 2019. Robust methods in mendelian randomization via penalization of heterogeneous causal estimates. PloS one **14**: e0222362.

[33] Saaristo T, Peltonen M, Lindström J, Saarikoski L, Sundvall J, Eriksson JG, and Tuomilehto J. 2005. Cross-sectional evaluation of the finnish diabetes risk score: a tool to identify undetected type 2 diabetes, abnormal glucose tolerance and metabolic syndrome. Diabetes and vascular disease research **2**: 67–72.

[34] Sanderson E, Davey Smith G, Windmeijer F, and Bowden J. 2019. An examination of multivariable mendelian randomization in the single-sample and two-sample summary data settings. International journal of epidemiology **48**: 713–727.

[35] Schalkamp AK, Peall KJ, Harrison NA, and Sandor C. 2023. Wearable movement-tracking data identify parkinson's disease years before clinical diagnosis. Nature Medicine **29**: 2048–2056.

[36] Shipe ME, Deppen SA, Farjah F, and Grogan EL. 2019. Developing prediction models for clinical use using logistic regression: an overview. Journal of thoracic disease **11**: S574.

[37] Spiller W, Hartwig FP, Sanderson E, Davey Smith G, and Bowden J. 2022. Interaction-based mendelian randomization with measured and unmeasured gene-by-covariate interactions. Plos one **17**: e0271933.

[38] Spooner A, Chen E, Sowmya A, Sachdev P, Kochan NA, Trollor J, and Brodaty H. 2020. A comparison of machine learning methods for survival analysis of high-dimensional clinical data for dementia prediction. Scientific reports **10**: 20410.

[39] Staley JR and Burgess S. 2017. Semiparametric methods for estimation of a nonlinear exposure-outcome relationship using instrumental variables with application to mendelian randomization. Genetic epidemiology **41**: 341–352.

[40] Verbanck M, Chen CY, Neale B, and Do R. 2018. Detection of widespread horizontal pleiotropy in causal relationships inferred from mendelian randomization between complex traits and diseases. Nature genetics **50**: 693–698.

[41] Wang J, Zhao Q, Bowden J, Hemani G, Davey Smith G, Small DS, and Zhang NR. 2021. Causal inference for heritable phenotypic risk factors using heterogeneous genetic instruments. PLoS genetics **17**: e1009575.

[42] Wang S and Kang H. 2022. Weak-instrument robust tests in two-sample summary-data mendelian randomization. Biometrics **78**: 1699–1713.

[43] Willetts M, Hollowell S, Aslett L, Holmes C, and Doherty A. 2018. Statistical machine learning of sleep and physical activity phenotypes from sensor data in 96,220 uk biobank participants. Scientific reports **8**: 1–10.

[44] Wu Y, Byrne EM, Zheng Z, Kemper KE, Yengo L, Mallett AJ, Yang J, Visscher PM, and Wray NR. 2019. Genome-wide association study of medication-use and associated disease in the uk biobank. Nature communications **10**: 1891.

[45] Ye T, Shao J, and Kang H. 2021. Debiased inverse-variance weighted estimator in two-sample summary-data mendelian randomization. The Annals of statistics **49**: 2079–2100.

[46] Zhao Q, Wang J, Hemani G, Bowden J, and Small DS. 2020. Statistical inference in two-sample summary-data mendelian randomization using robust adjusted profile score .

[47] Zhu Z, Zheng Z, Zhang F, Wu Y, Trzaskowski M, Maier R, Robinson MR, McGrath JJ, Visscher PM, Wray NR, et al.. 2018. Causal associations between risk factors and common diseases inferred from gwas summary data. Nature communications **9**: 1–12.
